# Toward Quantitative Models in Safety Assessment: A Case Study to Show Impact of Dose–Response Inference on hERG Inhibition Models

**DOI:** 10.3390/ijms24010635

**Published:** 2022-12-30

**Authors:** Fjodor Melnikov, Lennart T. Anger, Catrin Hasselgren

**Affiliations:** Department of Safety Assessment, Genentech, Inc., South San Francisco, CA 94080, USA

**Keywords:** hERG, computational toxicology, xgboost, data inference, safety assessment, drug development, dose–response, cardiotoxicity, QSAR

## Abstract

Due to challenges with historical data and the diversity of assay formats, in silico models for safety-related endpoints are often based on discretized data instead of the data on a natural continuous scale. Models for discretized endpoints have limitations in usage and interpretation that can impact compound design. Here, we present a consistent data inference approach, exemplified on two data sets of Ether-à-go-go-Related Gene (hERG) K+ inhibition data, for dose–response and screening experiments that are generally applicable for in vitro assays. hERG inhibition has been associated with severe cardiac effects and is one of the more prominent safety targets assessed in drug development, using a wide array of in vitro and in silico screening methods. In this study, the IC_50_ for hERG inhibition is estimated from diverse historical proprietary data. The IC_50_ derived from a two-point proprietary screening data set demonstrated high correlation (R = 0.98, MAE = 0.08) with IC_50s_ derived from six-point dose–response curves. Similar IC_50_ estimation accuracy was obtained on a public thallium flux assay data set (R = 0.90, MAE = 0.2). The IC_50_ data were used to develop a robust quantitative model. The model’s MAE (0.47) and R^2^ (0.46) were on par with literature statistics and approached assay reproducibility. Using a continuous model has high value for pharmaceutical projects, as it enables rank ordering of compounds and evaluation of compounds against project-specific inhibition thresholds. This data inference approach can be widely applicable to assays with quantitative readouts and has the potential to impact experimental design and improve model performance, interpretation, and acceptance across many standard safety endpoints.

## 1. Introduction

Drug development typically incorporates variety of in vitro assays to assess off-target interactions that may ultimately result in toxicity. Often, these assays are run in multiple formats depending on the stage of development, and the historical data for the same endpoint can originate from somewhat variable protocols. Such data lend themselves well for the development of in silico models that can be used to screen virtual molecules for series selection or to prioritize compounds for experimental follow-up within a chemical series. One drawback in this context is that data are often limited, and it is advantageous to combine data from a variety of experimental protocols for the same endpoint to maximize chemical space coverage. This practice typically leads to data discretization and the development of categorical models that can be used as coarse filters but may not necessarily provide more detailed output to the user.

A typical safety concern for pharmaceutical projects is the propensity for cardiac toxicity. Cellular action potential and the electrical activity of the heart are largely regulated by voltage-gated ion channels [1]. The human ether-a-go-go-related gene (*hERG* or *KCNH2*) encodes a K+ channel (Kv11.1 or hERG) that is responsible for rapid repolarization. It is essential for a standard electrocardiogram readout and normal heart function [2,3,4]. hERG inhibition may cause long QT syndrome (LQTS), arrhythmia, and Torsade de Pointes (TdP), which can lead to palpitations, fainting, seizures, and, in severe cases, even sudden death [2,5,6,7]. Structurally diverse compounds have been shown to inhibit the hERG ion channel, and a number of drugs from across therapeutic areas have been withdrawn from the market or severely restricted due to hERG-related cardiotoxicity [1,8], for example, astemizole, cisapride, terfenadine, vardenafil, and ziprasidone [9,10,11].

Given the severity of cardiac events related to hERG inhibition, early assessment of hERG-related cardiotoxicity has been an important step in drug development since the early 2000s [12]. The United States Food and Drug Administration (FDA) and the International Conference on Harmonization of Technical Requirements for Registration of Pharmaceuticals for Human Use (ICH) added safety criteria guidance for possible or high risk TdP to new drug applications [13,14]. All drug candidates are required to be screened for hERG liabilities prior to clinical trials and regulatory review. Consequently, early hERG inhibition evaluation has been widely adopted across the pharmaceutical industry to eliminate compounds with higher hERG inhibition potential [15,16]. Many in vitro screening methods for hERG inhibition are available, but the patch-clamp electrophysiological assay is the most widely used method for in vitro hERG inhibition assessments [17,18,19]. While the technology is improving, it is relatively costly, laborious, and time-consuming [20]. However, the accumulation of hERG inhibition data enabled the development of in silico screening methods. In silico tools allow for rapid and cost-effective hazard identification, and no chemical matter is required for in silico assessment, allowing chemists to evaluate theoretical compounds before synthesis. Thus, in silico tools for hERG assessment provide valuable insights during lead findings and optimization stages of drug development.

Many models for hERG inhibition have been developed over the past twenty years [11,15,16,21,22,23,24,25,26,27,28,29,30,31,32,33]. As in all applications, model quality and utility are largely dependent on the training data [34]. Older hERG modeling efforts were limited by small qualitative or quantitative data sets with limited validation [15]. The majority of the recent models focused on classifying hERG inhibition at 10 μM or at multiple thresholds [11,15,16,21,24,25,27,28,29,30,31]. While categorical models are useful for hazard identification at a specific threshold, they do not allow scientists to rank-order compounds and do not support a project-specific threshold without retraining. Furthermore, toxicologists typically interpret experimental hERG inhibition results in terms of quantitative IC_50_ values and often prefer models with outputs in the same format. Consequently, it is advisable to develop continuous hERG inhibition models instead of categorical models whenever possible to facilitate model acceptance. However, quantitative model development has been hindered by data challenges. Much hERG inhibition data from early project stages may come from non-ICH experimental protocols. These protocols may differ widely across projects, across industries, and over time, making it more difficult to compare results and develop a comprehensive data set. Many early hERG inhibition screening assays are run in one or two points and do not provide IC_50_ estimates. Screening compounds in one or two concentrations only is common practice for financial reasons [35]. In addition, when the IC_50_ values lie outside the range of tested concentration, the IC_50_ may be assigned to a constant with a “<” or “>” qualifier and cannot be used directly for quantitative modeling [33].

In this paper, we demonstrate the impact of historical data processing on model performance. More specifically, robust dose–response inference from historical data can enable continuous hERG inhibition models that predict pIC_50_s instead of activity categories. The model’s training set incorporated historical data from dose–response and two-point screening experiments. We used a robust dose–response inference approach to derive IC_50_s from the two-point screening data and incorporated the IC_50_ data into the continuous model development, substantially increasing the size of the data set and enhancing the model performance. We demonstrate the benefits of the quantitative approach and discuss its practical uses in drug development. Finally, we show that the two-point inference can be applied to public data sets such as the thallium flux assay data available in PubChem.

## 2. Results

### 2.1. Two-Point Data Inference

**Figure 1:**
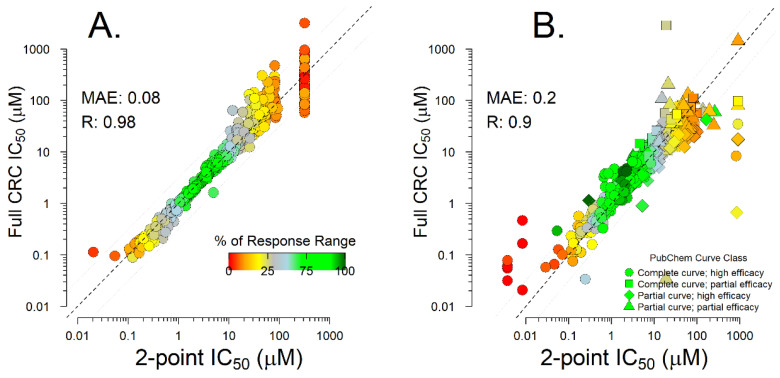
Correlation between IC_50_ estimates derived from full dose–response curves and two-point subsets of the same dose–response curves for in-house patch clamp data (**A**) and PubChem thallium flux assay data (**B**).

Historical data for hERG inhibition and other toxicology endpoints often come from experiments that differ in screening format or protocols. It is often difficult to extract consistent and reliable quantitative potency estimates from different types of experiments. Conventionally, most results from one- and two-point screening are discretized to binary categories. Consequently, models based on these data tend to predict binary activity instead of continuous potency. We used simplified dose–response inference based on a one-parameter hill equation (Equation (1)) to obtain consistent IC_50_ estimates from two-point hERG screening experiments. To ensure model quality, we validated the reliability of IC_50_ estimates derived from two-point-screening experiments by comparing IC_50_ derived from 4–15 concentration dose–response curves to IC_50_s derived from a two-concentration subset of the curves, as discussed in the Methods section. To increase confidence, the analysis was repeated in two distinct data sets: internal proprietary data from hERG patch clamp assay [36,37,38] and a public thallium flux assay available in PubChem [11,39]. IC_50_s derived from two-point subsets of the full dose–response series demonstrated excellent correlation with IC_50_s derived from the full titration series. The correlation was better for the path clamp assay data (R = 0.98, MAE = 0.08 log_10_ units, Figure 1A), and somewhat worse for thallium flax assay data (R = 0.90, MAE = 0.2 log_10_ units, Figure 1B). In both cases, the uncertainty derived from mathematical extrapolation outside the tested concentration ranges was below the uncertainty resulting from repeated experiments, which was 0.21 log_10_ units based on the in-house patch clamp data set. The PubChem thallium flux assays did not contain any biological replicates to assess experimental variability. Larger differences in IC_50_ estimates were associated with lower response (% response range) captured in the two selected concentrations. Extreme errors in the thallium flax assay estimates were associated with non-monotonic responses and similar data idiosyncrasies, where the compounds produced no inhibition at the two concentrations selected for two-point screening but inhibited hERG at lower concentrations (see Figure S1 for examples).

### 2.2. hERG Model Performance

We compared four modeling strategies to evaluate the impact of the data processing on model performance. Two models for categorical and two models for continuous outcomes were developed (Table 1). The expanded continuous data (ECD) model included the pIC_50_s extrapolated from two-point hERG screening data, while the limited continuous data (LCD) model was based on the pIC_50_s derived from traditional dose–response experiments only. For categorical models, the data were discretized at a 10 μM threshold as discussed in the Methods section. The all binary data (ABD) model included data for all compounds that could be discretized at the 10 μM threshold without ambiguity. The high confidence binary data (HCBD) model only included compounds with % inhibition < 30% or >70% at 10 μM to minimize the influence of experimental variability on the classification.

**Figure 2:**
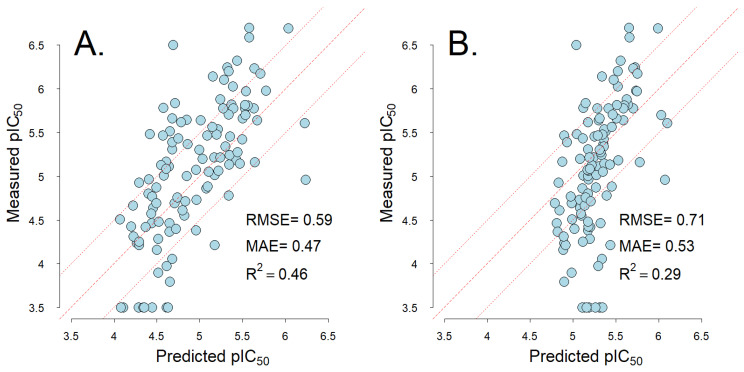
The relationship between predicted and measured pIC_50_ hERG inhibition estimates. The predictions are from the ECD model (**A**) and LCD model (**B**). The dashed line shows one-to-one correlation and the dotted lines mark 0.3 pIC_50_ errors around one-to-one correlation.

The expansion of the data set with pIC_50_s derived from two-point experiments and estimates outside the tested concentration ranges improved the empirical model performance (Figure 2). The ECD model demonstrated lower RMSE, lower MAE, higher R^2^ and ΔQ_2_ compared to the LCD model (Table 2). Notably, these results are not consistent with the internal validation. In the five-fold internal cross-validation, the LCD model showed lower MAE (0.25) than the ECD model (MAE = 0.34) (Table 3).

Since the data for development of the categorical models were discretized at the 10 μM threshold, we discretized the predictions made by the ECD and LCD models around the same 10 μM threshold to compare model performance. The ECD model demonstrated higher sensitivity, balanced accuracy, NPV, and PPV (Table 2). Notably, the LCD model showed higher sensitivity and NPV than all other models but had the lowest balanced accuracy. The two categorical models performed comparably on the prospective test set (Table 2). The ABD model showed higher sensitivity, NPV, but lower balanced accuracy, specificity, and NPV compared to the HCBD model. Of the two categorical models, the ABD model showed better balanced accuracy during cross-validation; 0.90 vs. 0.83 for ABD and HCBD models, respectively (Table 3). Importantly, the ECD model showed the highest improvement over random accuracy (ΔQ_2_ = 0.28), compared to ABD and HCBD models (ΔQ_2_ = 0.21). Similar trends were observed when assessing model performance on a highest confidence test subset, where observations sufficiently close to the 10 μM threshold were excluded to account for experimental uncertainty (Table 2). Overall, the ECD model demonstrated the highest BA, Q_2_, ΔQ_2,_ and PPV, as well as higher sensitivity and NPV than the categorical models. The ABD model showed higher sensitivity, NPV, but lower balanced accuracy, specificity, and NPV compared to the HCBD model. Table S3 provides confusion matrix statistics for each of the four models.

### 2.3. Alternative Decision Thresholds

Continuous models allow users to set different decision thresholds without retraining the model. We evaluated the more robust ECD model on four activity thresholds that are of typical interest to drug development projects (Table 2). As expected, model performance trended with the activity distribution in the data set. The highest balanced accuracy (BA = 0.79) was observed at the 10 μM threshold, where the fraction of active hERG inhibitors approached 0.5 (prevalence = 0.58). While sensitivity varied substantially across thresholds, the NPV, PPV and BA remained more consistent across the threshold range.

## 3. Discussion

Learning from imprecise data has been a growing interest across computational sciences [40]. The topic is of particular interest in computational toxicology since model development in the field usually relies on historical data collected in diverse formats for different purposes and, frequently, from different experimental protocols. Furthermore, toxicological data sets are often small, ranging from hundreds to a few thousand observations. Thus, each data point could be critical for in silico model development. For example, to include the most data, many hERG inhibition models have been developed using a combination of diverse public and private historical data sets [11,15,16,21,22,23,24,25,26,27,28,29,30,31,32,33]. The majority of these models treated inhibition as a categorical outcome around a threshold, in part, due to high prevalence of ambiguous IC_50_s with “>” or “<” qualifiers. These data points are generated typically when the experimental IC_50_ is outside the range of tested concentrations and the curve-fitting algorithms do not extrapolate beyond the tested concentration range when determining the IC_50_. Categorical models typically ignored these data or assigned surrogate values. While literature precedents have suggested that the addition of imprecise data improves model performance, careful interpretation of the data uncertainty in the context of experimental protocol and assay details is essential [32,40,41,42]. Previous studies indicated that across secondary pharmacology safety screening targets, IC_50_s can be reliably estimated from single concentration data if the measured % inhibition falls between 20% and 80% [35]. Based on these hypotheses, we fit dose–response curves to all in-house patch-clamp data regardless of experimental format and extrapolated IC_50_ estimates beyond tested concentration ranges. This approach allowed us to use all data from the faster and cheaper two-point screening assay in a continuous model and expanded the internal data set more than 10-fold. IC_50_ values from the two-point screening data accurately estimated IC_50_s from more complete dose–response curves when the measured % inhibition fell between 20% and 90% (Figure 1). The median pIC_50_ errors due to mathematical extrapolation outside the tested concentration ranges for titration series with measured % inhibition between 20% and 90% ranged from 0.01 to 0.1 in the two data sets and were far below experimental uncertainty in repeated experiments (0.21 log_10_ units). Estimating IC_50_s from two-point screening was an effective strategy of obtaining reliable IC_50_ estimates and expanding modeling data sets for the hERG patch clamp and thallium flux assays. While the two-point inference based on the 1 and 10 μM concentrations worked well for a vast majority of experiments, the exact IC_50_s for very potent or inactive compounds may be difficult to ascertain when data are collected at these two concentration only. In the extreme cases, the data may provide very limited information about the shape of the dose–response curve and the IC_50_ location. Our analysis suggested that IC_50_s between 0.1 and 100 μM can be reliably estimated from these two concentrations (Figure 1); i.e., the extrapolation is robust within one log unit of the tested concentrations. However, when a more exact IC_50_ estimate for more potent or less potent compounds is required, additional or different concentrations should be screened. The 1 and 10 μM values were selected based on their relevance to decision boundaries for hERG inhibition. Practically, any compound that is too potent to have its IC_50_ reliably estimated on these two concentrations is unlikely to be a viable drug candidate due to hERG inhibition concerns. Conversely, compounds completely inactive up to 10 μM pose no concern based on this assay. Thus, as with most computational tasks, the experimental design still plays a critical role in final data quality and interpretability. While robust inference can help maximize the knowledge gained from data, the upper limit is still dictated by the choices made in the experimental design.

Finally, we note that while this approach works well for the two hERG assays discussed in the paper, it should be further validated when applied to new assays. This is particularly critical for endpoints that are more likely to exhibit non-monotonic responses, such as cytotoxicity. However, given the ample and growing interest in predictive models for drug discovery [43] and challenges associated with balancing categorical data sets [44], these data inference approaches can be of high interest for developing more robust models in computational toxicology and drug discovery.

Expanding the quantitative data set with IC_50_s derived from the two-point data helped develop a more robust, continuous hERG model for internal project use (Table 2). Data set composition has been shown to have large effects on the performance of categorical models [45]. The trend held true for both the categorical and the continuous models in our study. When used to classify compounds at the 10 μM threshold, the ABD model showed higher specificity and balanced accuracy but lower sensitivity as compared with the HCBD model that was built on fewer data points with higher classification confidence. These results were expected, as the infusion of extrapolated IC_50_s into the ABD model added 2250 compounds with IC_50_ values above 10 μM and 179 compounds with IC_50_ values below 10 μM, enhancing the specificity and PPV. Notably, the ECD model showed marginal improvement compared to the two classification models, when evaluated on its ability to classify compounds at the 10 μM threshold, and demonstrated notably higher improvement over random accuracy (Table 2). Finally, we noted that the common practice of removing compounds near the activity classification cutoff during model training had minimal influence on the model performance (Table 2). The practice is typically used to avoid including data points with ambiguous true classification in model training. However, in this case-study, this practice increased the apparent model performance during cross-validation only (Table 3) and did not have any notable impact when the performance was assessed on the external test set (Table 2). Our findings suggested that the efforts to be overly selective with the data included in the training set may lead to over-fitting and may inflate model confidence if the model is assessed by cross-validation alone. More generally, the observations support the ML hypothesis that additional data, even that of somewhat lower quality, may improve model performance when compared to models based on smaller data sets with the highest-quality data only [32,40,41,42].

The training and test sets differed substantially in the prevalence of hERG-inhibiting compounds (0.39 and 0.58, respectively). This difference provided an additional challenge for the model beyond the typically challenging prospective assessment. However, this set up presented a more realistic assessment of the model utility in the evolving pharmacological space. Although the statistics and model performance may be seen as suboptimal, these statistics provide a conservative assessment of model performance on new projects or new chemical series. The continuous model statistics compared favorably with recent large data hERG models. Ma et al. reported R^2^ values ranging from 0.305 to 0.352 for hERG inhibition models [33]. The ECD model had an R^2^ of 0.46 on the prospective test set. Radchenko et al. reported Q^2^ = 0.6 and RMSE_cv_ = 0.55 in a larger study that included cross-validation assessment only [46] In cross-validation, the ECD model was comparable. During hyperparameter optimization, the models exhibited Q^2^ values ranging from 0.66 to 0.69, and RMSE_cv_ ranging from 0.32 to 0.35. These differences in performance statistics between internal and external validation once again highlight the importance of external and prospective model assessments. Older studies have reported Q^2^ values as high as 0.9 on test sets under 14 compounds [15]. While these provide an upward bound of model potential, they are unlikely to perform equally well in changing chemical space. Models trained and tested on small data sets may not be adequate to address the challenges faced in an industrial setting, where the models are expected to make useful predictions on a constantly changing chemical.

The ECD model that predicted a continuous IC_50_ value demonstrated marginal improvement in performance statistics when used for classification at the 10 μM threshold and when compared to the two categorical models (Table 2). While it may be inappropriate to generalize findings in a purely statistical sense, models for continuous data present several practical advantages for drug development. Most importantly, continuous IC_50_ predictions can be used to rank-order compounds. The feature is particularly useful during the early stage of drug development, as it allows chemists to rank-order theoretical compounds before synthesis. The predictions can be used in multi-parameter optimization to select chemical space or series expected to be less likely to produce adverse effects down the road. Second, scientists may evaluate new compounds against different project-specific thresholds using the same model. The flexibility of ranking compounds based on IC_50_ predictions or choosing an activity threshold is particularly useful for promiscuous targets, which may be evaluated across many projects. Owing to the flexibility of the hERG ligand binding site, hERG is a promiscuous target that may be inhibited by structurally diverse substances [47]. Continuous models allow scientists to choose different thresholds based on the relative potency of the molecules within a specific project or a specific series. Theoretically, a series of binary models can be used to bin compounds to be more refined; however, practically, the modeling approach would be more difficult to execute and maintain. Model development would be substantially influenced by data set composition, as some bins may have few or no compounds. More conceptually, the models would discretize the data that are inherently continuous in nature, thus reducing the information each model can learn from. Finally, to maximize model acceptance, it is usually advisable to provide project teams with a single model output, so that outputs from multiple models would have to be further aggregated. IC_50_ is the standard and the most robust output of the hERG patch clamp and thallium flux assays and is the parameter most commonly used for interpretation by chemists and toxicologists. Consequently, it is advantageous to report predicted IC_50s_ to provide users with familiar and easily comparable values; thus further improving model acceptance.

In silico models that can reliably predict hERG blockade are useful to medicinal chemists and toxicologists during hit finding, hit-to-lead, and lead optimization stages of drug development. They allow projects to screen out potentially hazardous molecules early in the development process, saving time and money [48]. All models presented here showed high PPV and sensitivity at the 10 μM threshold (Table 2). These parameters are particularly useful in early drug development where it is important to avoid unnecessarily eliminating compounds, i.e., model predictions are unlikely to flag an inactive molecule as a hERG inhibitor. The predictions are validated with dose–response in vitro experiments later in the drug development process, and the early in silico profiling can help projects to selects leads with the best safety margins, rather than simply selecting those that have efficacy at the lowest plasma concentration. When applied to broader sets of industrial compounds, the in silico hERG models can help fill data gaps and guide risk minimization strategies.

One limitation of extracting IC_50_s from a one- or two-point screen in functional cell-based assays is the inability to capture partial activity. The models assumed 100% inhibition at some high concentration. While partial activity did not appear to contribute significantly to the types of responses observed in our data set, it may be a greater concern for other assays or in different chemical spaces. Analyzing and modeling different response patterns is a topic for further research.

Finally, hERG inhibition is important but not sufficient for predicting QT prolongation and drug-induced TdP. Some molecules may inhibit multiple ion channels with the cumulative effect, normalizing the standard depolarization–repolarization cycle [49]. Another advantage of the continuous modeling for cardiotoxicity prediction is that quantitative outputs from robust channel inhibition models can be combined with in silico reconstructions of cellular cardiac electrophysiological activity to model a more holistic output of overall cardiotoxicity potential based on chemical structure [50].

## 4. Materials and Methods

### 4.1. Chemical Data Sets

We compiled a data set of historic small molecules from Genentech pipeline with patch clamp hERG inhibition data. The compounds included 80 projects spanning the 2007–2021 time frame. The structures for all compounds used in the screening were standardized using OpenEye Scientific Software [51]. The counter ions were removed, and tautomeric forms standardized as discussed elsewhere [51,52]. The standardized structures were grouped based on InChIKey [53] calculated using RDKit toolbox [54] in Python v 3.7. All models were based on 2D descriptors, and hence, enantiomers and diastereomers appeared identical in the descriptor space, and single unique 2D structure was retained when enantiomers or diostereomers were present. A weighted average of pIC_50_ was calculated when multiple experiments were available for a 2D structure. The resulting data set contained 4200 unique 2D structures (Table S2).

### 4.2. Chemical Descriptors

We used a combination of molecular fingerprints and physicochemical properties to describe 2D chemical structures. Morgan (Circular), MACCs, Topological (RDKit), Feature Morgan, Pattern, Topological Torsion fingerprints and RDKit descriptors were calculated using RDKit toolbox [54] in Python v 3.7. In addition, Moka 2.6.5 [55] from Molecular Discovery was used to calculate pKa values, and internal models were used to produce additional substructure and partitioning descriptors [26,56]. The fingerprints and molecular descriptors were used to feature molecular structure for model development. The features were selected via regularization and feature selection filtering methods implemented in XGBoost.

### 4.3. Dose–Response Inference for Genentech Data

We collected 6216 historical experiments from the automated QPatch or SyncoPatch whole cell patch-clamp assay (Table S2) for the 4200 unique 2D structures [36,37,38]. The 6216 hERG patch-clamp experiments differed widely in the number of concentrations screened per titration series. The majority of the data were collected in two-point screening format, where compounds are screened at 1 and 10 μM. In all cases, the experiments are normalized to positive (E-4031) and negative (DMSO) controls. To ensure consistent inference across experiments, we developed an IC_50_ inference protocol that reliably extrapolates IC_50_s from two-point patch clamp screening data. In addition, we allowed the algorithm to extrapolate IC_50_s outside of the tested concentration ranges to support continuous model development. All titration series were fit to a simplified hill curve by fixing three of the four hill curve parameters (Equation (1)). The upper asymptote was set to 100% inhibition, i.e., the inhibition observed in the positive control (E-4031). The lower asymptote of the hill curve was fixed to 0% inhibition, i.e., normalized response observed in DMSO negative controls. The hill slope was set to 1. The three constant parameters were derived via a combination of heuristic information about the experimental set up and empirical data analysis. The resulting 1-parameter hill curves were fit in R statistical environment [57] using a likelihood-based optimization routine via Nelder–Mead optimization method as previously discussed [41,58].

(1) $$\%\;\mathrm{Inhibition}=\frac{100}{1+\frac{{\mathrm{IC}}_{50}}{\left[\mathrm{concentration}\right]}}$$

**Figure 3:**
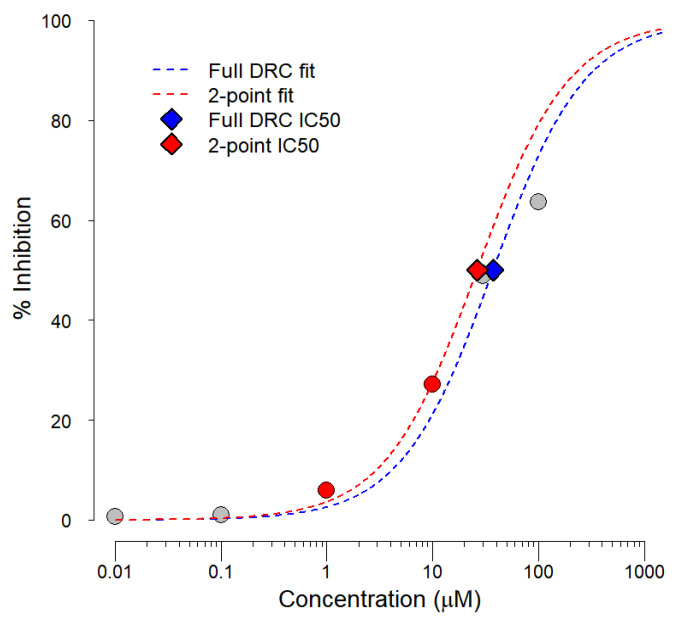
Sample plot for IC_50_ derivation from six-point and two-point titration series.

Since 85% of all data were generated in two-point-screening format, we ensured that the IC_50_ estimates from the two-point experiments were reliable. To assess the reliability of two-point IC_50_ estimates, we compared IC_50_ estimates from titration series with more than two points to the IC_50_s derived from two-point data sets in the same experiment (Figure 3). The 1 and 10 μM concentrations were selected as the two-point subsets of dose–response curves because these two concentrations are screened in all two-point experiments and have direct relevance for decision thresholds in drug safety.

In all cases, the curves were fit when at least one response in a titration series exceeded 10% inhibition. When no curve could be fit to a titration series due to lack of response in all tested concentrations, the IC_50_ was assigned to 1.5 log units above the highest tested concentration. The approach produced IC_50_ estimates of 316 μM for a typical experiment where a compound was tested up to 10 μM and hERG inhibition did not exceed 10%. The standard value was selected based on empirical evidence and the typical shape of the dose–response curves in the assays.

As discussed above, the data were combined using 2D chemical structures as unique identifiers. When pIC_50_ values from multiple experiments were available for a 2D structure, we calculated a weighted average of the pIC_50_ values. The weights were calculated based on the response range (Equation (3)), i.e., the maximum range of % inhibition response observed across the tested concentrations in each titration series. Finally, the IC_50_ values were converted to pIC_50_ for modeling effort (Equation (3))

(2) Response Range=max(100−min(% Inh), max(% Inh))

(3) $${\mathrm{pIC}}_{50}=-\log_{10}\left({\mathrm{IC}}_{50}\right)$$

To demonstrate the applicability of the approach to other data, we applied the same data inference algorithm to the public thallium flux assay data. The thallium flux assay detects inhibition of the hERG channels by measuring flow of a surrogate ion, thallium, in a homogenous assay format. It is a high-throughput alternative to the more expensive patch clamp technology that has been extensively validated on small molecules with the potential to block hERG and induce LQTS [59,60]. We extracted data for 5281 dose–response experiments from PubChem (AID 588834) on 1 August 2022. The experiments were run in 7, 14, or 15 concentrations ranging from 0.1 nM to 92 μM. The curves were categorized into several classes based on the fraction of response achieved in each curve and the quality of the dose–response fit [61]. To enable reliable comparison metrics, we excluded curves annotated with poor fits, curves with no response across the tested concentration (PubChem inactive), and curves designated as “single point of activity”. The remaining data set contained 629 experiments. To assess the utility of the two-point inference, we selected the two concentrations closest to 1 and 10 μM, which were 0.84 and 9.2 μM in this data set. We then fit dose–response curves to the full concentration response curves and the two-point subsets of the data as discussed above.

### 4.4. hERG Modeling Strategies

To assess the impact of data inference on model performance and utility, we developed four models that reflect common data processing and model development practices in computational toxicology (Table 1). Two continuous models aimed to predict pIC_50_ (ECD and LCD), and two discretized the pIC_50_ values into active and inactive at the 10 μM threshold (ABD and HCBD). The 10 μM threshold was chosen based on common hERG data guidance in safety assessment and literature precedence [16,21,24,31]. The ECD model incorporated all available data. The LCD model only included data points with IC_50_ values within the tested concentration ranges. The ABD model included data with congruent classification at the 10 μM threshold. Data were excluded from the ABD training set if multiple experiments showed IC_50_ values above and below the 10 μM threshold for the same compound. HCBD model included only the data with higher classification confidence at the 10 μM threshold. In addition, compounds with % inhibition > 30% and <70% at 10 μM were designated as ambiguous for the purposes of classification and were excluded from the HCBD model training set. The HCBD model was included to assess the impact of the common categorical modeling practice where scientists exclude compounds near the activity threshold from the training set [62].

### 4.5. Statistical Methods

All four models were trained using eXtreme Gradient Boosting (XGBoost) implementation in the R package XGBoost [63]. The boosted decision tree approaches have demonstrated good results across cheminformatics and broader modeling tasks [16,64]. This modeling method has several advantages, including improved model performance, automatic feature selection, and interpretable output. Hyperparameters (Table S1) and final model features were elected by cross-validation. The final features for each model were selected via combination of regularization and feature selection hyperparameters in the XGBoost algorithm.

For all models, the data sets were split into training and prospective test sets before any further data processing. To mimic industrial use, the test set was selected on prospective bases, i.e., the set of 115 compounds most recently screened in 2021. The representations of the chemical space for test and train sets based on common dimension reduction techniques [65,66,67,68] are provided in Figure S2. Linear combinations of features in the training set were removed and hyperparameters were optimized using 5-fold and 8-fold nested cross-validation. The hyperparameters for each model are available in Table S1. XGBoost models with the optimized hyperparameters were built on the entire training set and evaluated on the prospective test set. The results of 5-fold cross-validation for each model are provided in Table 3.

### 4.6. Model Performance

A single prospective test set was used to assess and compare model performance. We used a comprehensive set of evolution metrics to assess model performance on the prospective test set (N = 115). These included sensitivity (Sens), specificity (Spec), positive predictive value (PPV), negative predictive value (NPV), balanced accuracy (BA), and accuracy (Q_2_) [16]. In addition, we included random accuracy (Q_2,rnd_) and model improvement over random accuracy (ΔQ_2_) for alternative metrics of model performance [69]. Since experimental variability may render classification around a threshold ambiguous, we identified a subset of the test data with higher experimental classification confidence for model evaluation. A subset included compounds with pIC_50_ values between 4.8 and 5.2 (N = 94). pIC_50_ values within 0.2 log units of the 10 μM threshold were removed based on the background assay variability: the median difference between IC_50_ values across experiments was 0.21 log units.

## 5. Conclusions

In this paper, we outlined the impact of historical data processing on model development, using hERG patch clamp inhibition assay as a case study. We demonstrated that pIC_50_ derived from two-point screening data can serve as a robust estimate for pIC_50_s derived from six-point dose–response curves, especially within the concentration ranges that are typically relevant for drug development decisions. Quantitative pIC_50_ inference from screening data enable the development of a quantitative QSAR model that can predict pIC_50_s for hERG inhibition instead of activity categories. This model performed on par with categorical models and demonstrated higher improvement over random accuracy when the output is discretized to predict activity categories. Furthermore, the continuous model enabled scientists to rank order and prioritize compounds in early research or to classify compounds around project-specific thresholds without retraining models. The pIC_50_ inference approach used here can be applied to expand the development of quantitative models in computational toxicology and drug development. It may be particularly impactful when modeling data that are highly categorically imbalanced. However, we advise scientists to review the pIC_50_ extrapolation results when applying similar approaches to new assays, as the inference can be complicated by non-monotonic dose–response curves or noisy assay data.

## Figures and Tables

**Table 1:** Model training set summary.

Model	Type	N	Data Included
Expanded continuous data (ECD)	Continuous	4081	All pIC_50_s from all experiments, including pIC_50_s extrapolated outside of the tested concentration ranges
Limited continuous data (LCD)	Continuous	1686	pIC_50_s from traditional dose–response experiments, calculated without extrapolation
All binary data (ABD)	Categorical	3903	All compounds for which consistent classification at the 10 μM threshold; prevalence = 40.9%
High confidence binary data (HCBD)	Categorical	2812	All compounds with % inhibition < 30% or >70% at 10 μM; prevalence = 35.9%

N—number of compounds in the model’s training set. Prevalence values are reported for categorical data sets around the 10 μM threshold.

**Table 2:** Model performance statistics.

Model	Ac.Thr.	Test Set	Sens	Spec	PPV	NPV	Prev	BA	Q_2_	Q_2,rnd_	ΔQ_2_
ECD	10	All	0.69	0.90	0.90	0.67	0.58	0.79	0.77	0.49	0.28
LCD	10	All	0.96	0.29	0.65	0.82	0.58	0.62	0.68	0.56	0.12
ABD	10	All	0.58	0.83	0.83	0.59	0.58	0.71	0.69	0.48	0.21
HCBD	10	All	0.54	0.90	0.88	0.58	0.58	0.72	0.69	0.48	0.21
ECD	10	HC	0.74	0.95	0.95	0.74	0.56	0.84	0.83	0.49	0.34
LCD	10	HC	0.96	0.32	0.65	0.87	0.56	0.64	0.68	0.54	0.14
ABD	10	HC	0.64	0.85	0.85	0.65	0.56	0.75	0.73	0.49	0.24
HCBD	10	HC	0.58	0.93	0.91	0.63	0.56	0.76	0.73	0.48	0.25
ECD	1	All	0.08	0.98	0.33	0.90	0.10	0.53	0.89	0.88	0.01
ECD	3	All	0.40	0.98	0.88	0.79	0.30	0.69	0.80	0.64	0.16
ECD	5	All	0.51	0.88	0.75	0.72	0.41	0.70	0.73	0.54	0.19
ECD	30	All	0.88	0.62	0.87	0.64	0.75	0.75	0.82	0.63	0.19

Model—model number as described in Table 1. Ac.Thr.—activity threshold for the model assessment (μM). Prev.—prevalence of active cpds at the activity threshold. HC—test set with observations at 10 μM threshold removed; contains data with 4.8 > pIC_50_ > 5.2. Sens—sensitivity, Spec—specificity, PPV—positive predictive value, NPV—negative predictive value, Prev—prevalence of positive classification around the activity threshold, BA—balanced accuracy, Q_2_—accuracy, Q_2,rnd_—random accuracy = [(TP + FN) (TP + FP) + (TN + FN) (TN + FP)] /N^2^, ΔQ_2_ = Q_2_ − Q_2,rnd_.

**Table 3:** Results for the internal cross-validation.

Model	Metric ^1^	Iteration	Value
ECD	MAE	1	0.334
ECD	MAE	2	0.347
ECD	MAE	3	0.353
ECD	MAE	4	0.338
ECD	MAE	5	0.341
**ECD**	**MAE**	**avg**	**0.343**
LCD	MAE	1	0.253
LCD	MAE	2	0.243
LCD	MAE	3	0.253
LCD	MAE	4	0.259
LCD	MAE	5	0.257
**LCD**	**MAE**	**avg**	**0.253**
ABD	BA	1	0.814
ABD	BA	2	0.824
ABD	BA	3	0.838
ABD	BA	4	0.846
ABD	BA	5	0.835
**ABD**	**BA**	**avg**	**0.831**
HCBD	BA	1	0.882
HCBD	BA	2	0.915
HCBD	BA	3	0.881
HCBD	BA	4	0.911
HCBD	BA	5	0.899
**HCBD**	**BA**	**avg**	**0.898**

^1^—Measure used to assess model performance in five-fold CV; MAE—mean absolute error. BA—Balanced accuracy.

## Data Availability

R, MLR, and XGBoost are freely available from https://cran.r-project.org/ on 2 August 2022. HERG patch clamp data and XGBoost model parameter sets for all models are provided as part of the Supporting Information. Chemical structures for internal molecules could not be disclosed. The thallium flux assay data are available from PubChem AID 588834 (https://pubchem.ncbi.nlm.nih.gov/bioassay/588834, accessed on 2 August 2022).

## Supplementary Materials

The following supporting information can be downloaded at: https://www.mdpi.com/article/10.3390/ijms24010635/s1, Table S1: Optimized hyperparameters used by each of the four models; Table S2: Genentech hERG data; Table S3: Confusion matrix statistics; Figure S1: Dose–response plots for PubChem data; Figure S2: Chemical space representation for compounds in the data set.
