# Supplementary material for: Toward Quantitative Models in Safety Assessment: A Case Study to Show Impact of Dose–Response Inference on hERG Inhibition Models
Source: Int J Mol Sci. 2022 Dec 30;24(1):635. doi: 10.3390/ijms24010635 (PMC9820331; doi:10.3390/ijms24010635)
Supplement: Supplementary file 1 [file ijms-24-00635-s001.zip › Tables S1 and S3, Figure S2.pdf]

Supplementary Information for

Toward Quantitative Models in Safety Assessment: A Case Study to Show Impact of Dose-Response Inference on hERG Inhibition Models

Fjodor Melnikov [1,*], Lennart T. Anger [1], Catrin Hasselgren [1]

1 Department of Safety Assessment, Genentech, Inc., South San Francisco, CA 94080, USA.

Fjodor Melnikov; ORCID: 0000-0003-3743-1376
* Correspondence: melnikof@gene.com

This PDF file includes:

Table S1 - Optimized hyperparameters used by each of the four models
Table S3 – Confusion matrix statistics
Figure S2 – Chemical space representation for compounds in the data set

Other supplementary materials for this manuscript include the following:

Table S2 – Modeling dataset
Figure S1 - Dose-response plots for Pubchem data

Table S1. Optimized hyperparameters used by each of the four models.

	Number of XGB Rounds	***Gamma***	***Max. depth***	***Minimum child weight***	***eta***	***Fraction of variables sampled by tree***
Model 1	201	0.56	12	6	0.12	0.63
Model 2	227	0.44	9	5	0.12	0.52
Model 3	160	0.25	20	2	0.25	0.7
Model 4	200	0.2	20	3	0.25	0.66

Table S3. Confusion matrix statistics

	TP	***FN***	***FP***	***TN***
ECD	46	21	5	43
LCD	64	3	34	14
ABD	39	28	8	40
HCBD	36	31	5	43

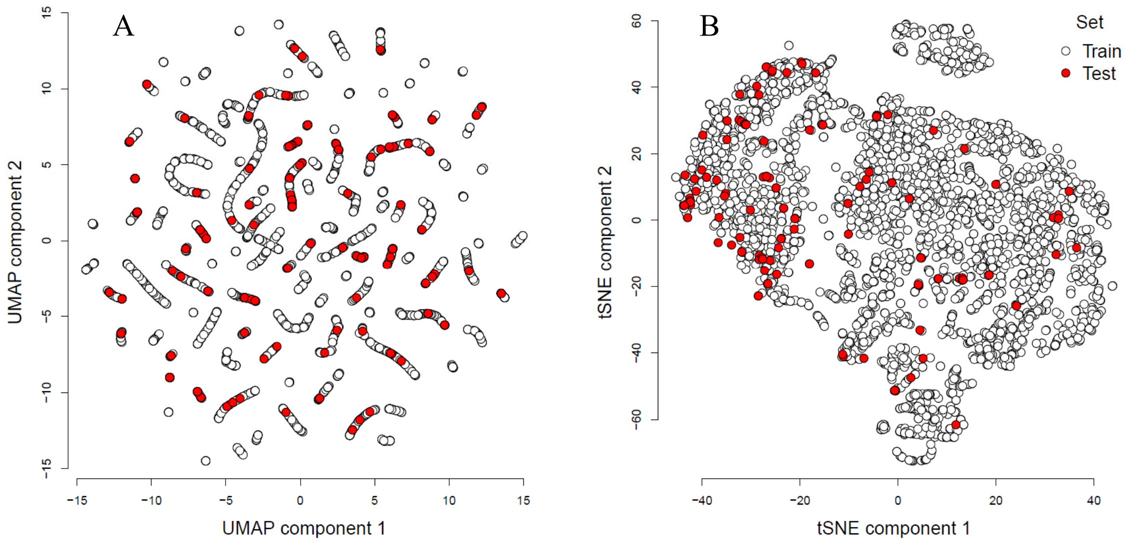

Figure S2: Chemical space representation for compounds in the data set using two dimension reduction techniques: Uniform Manifold Approximation and Projection (UMAP) (A), and t-distributed stochastic neighbor embedding (t-SNE) (B). The UMAP[1] embedding was calculated using R package umap[2], and the t-SNE[3] representation was calculated using the R package rtsne[4] with default parameters.

References:

1. McInnes, L.; Healy, J.; Melville, J. UMAP: Uniform Manifold Approximation and Projection for Dimension Reduction 2020.
2. Konopka, T. _umap: Uniform Manifold Approximation and Projection_. R Package Version 0.2.9.0, <https://CRAN.R-Project.Org/Package=umap>. 2022.
3. Maaten, L.V.D.; Hinton, G.E. Visualizing Data Using T-SNE. *Journal of Machine Learning Research* **2008**.
4. Krijthe, J. Rtsne: T-Distributed Stochastic Neighbor Embedding Using a Barnes-Hut Implementation, URL: Https://Github.Com/Jkrijthe/Rtsne 2015.
