# Supplementary figures and images for: Toward Quantitative Models in Safety Assessment: A Case Study to Show Impact of Dose–Response Inference on hERG Inhibition Models
Source: Int J Mol Sci. 2022 Dec 30;24(1):635. doi: 10.3390/ijms24010635 (PMC9820331; doi:10.3390/ijms24010635)

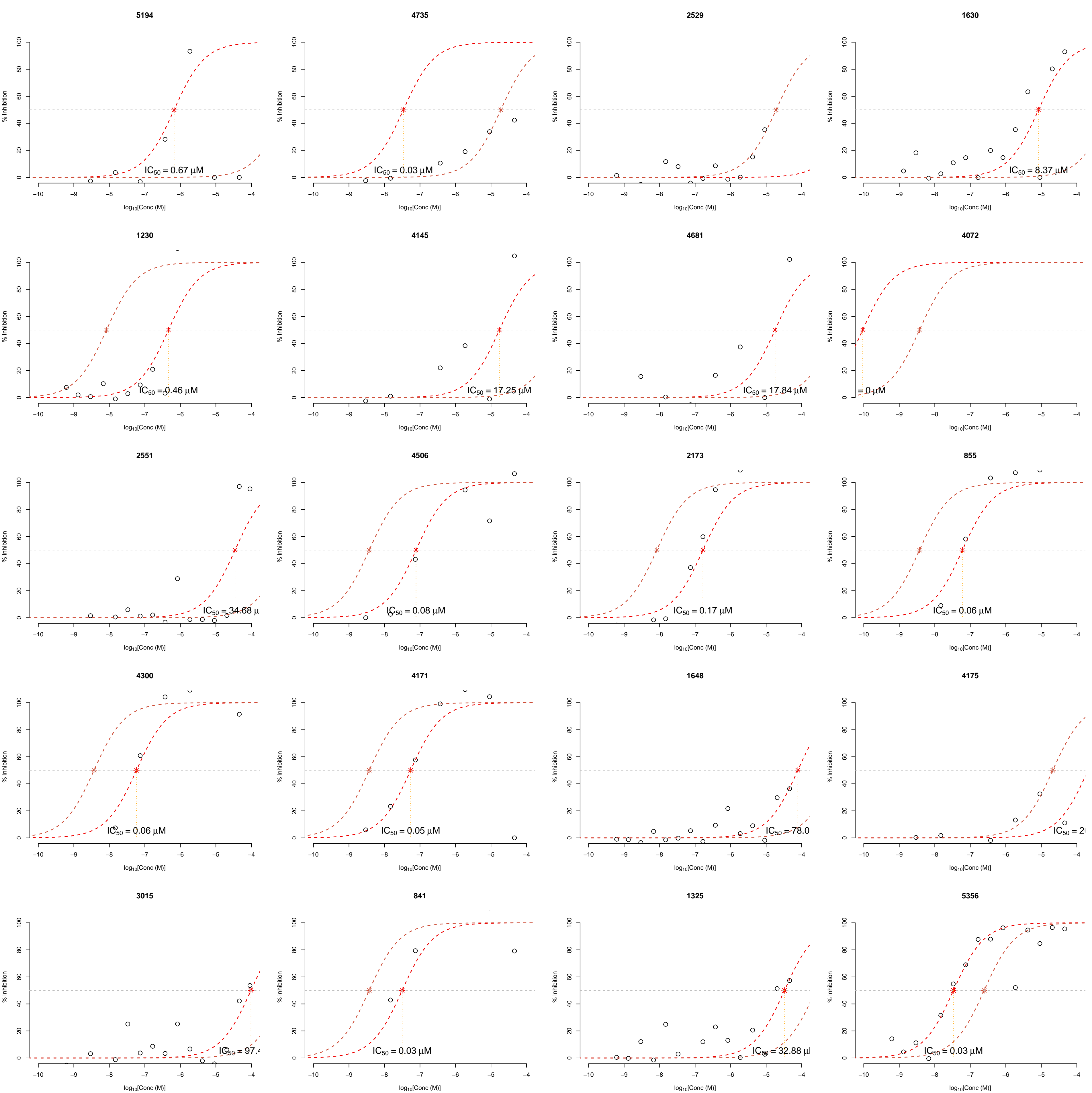

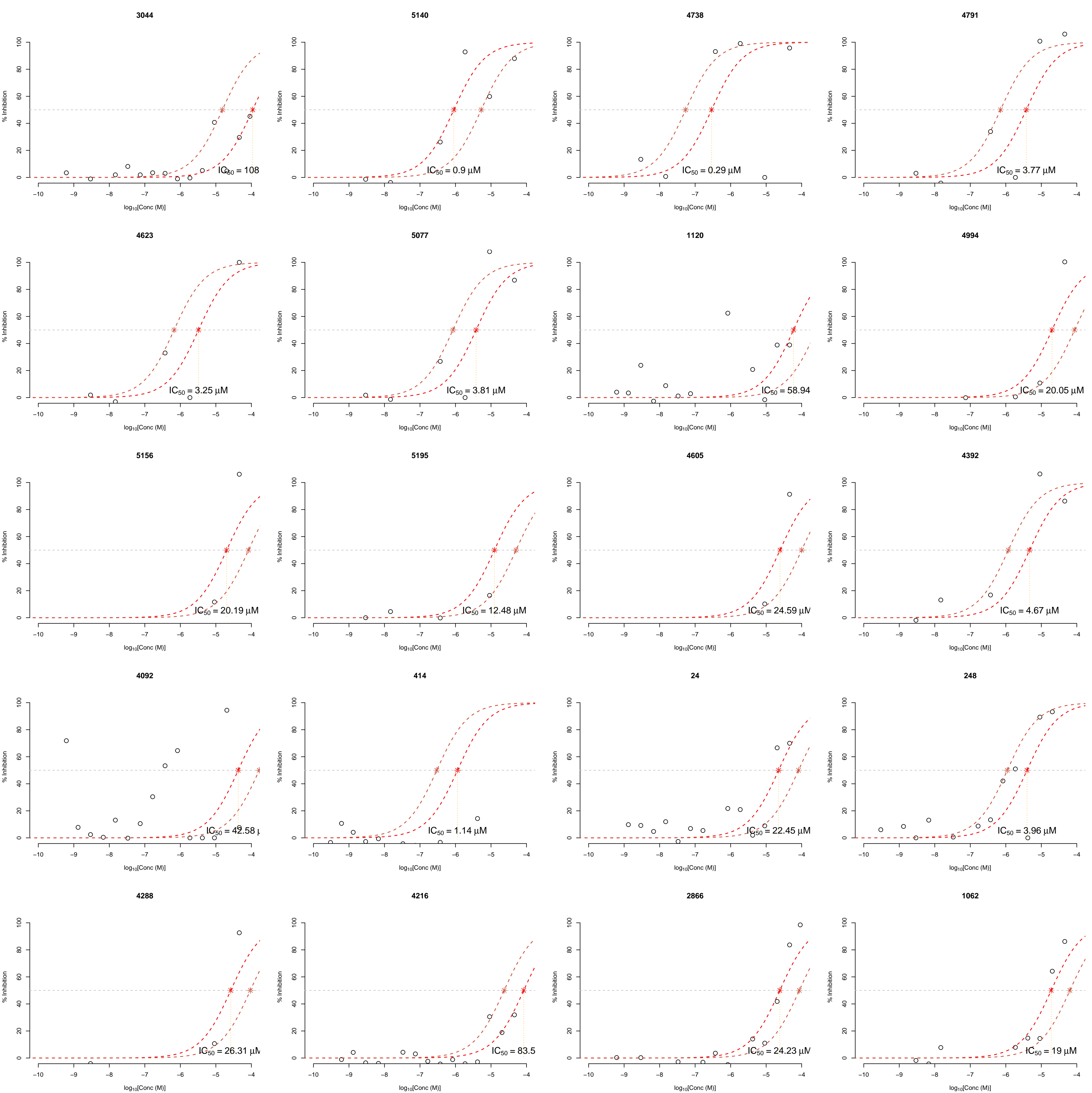

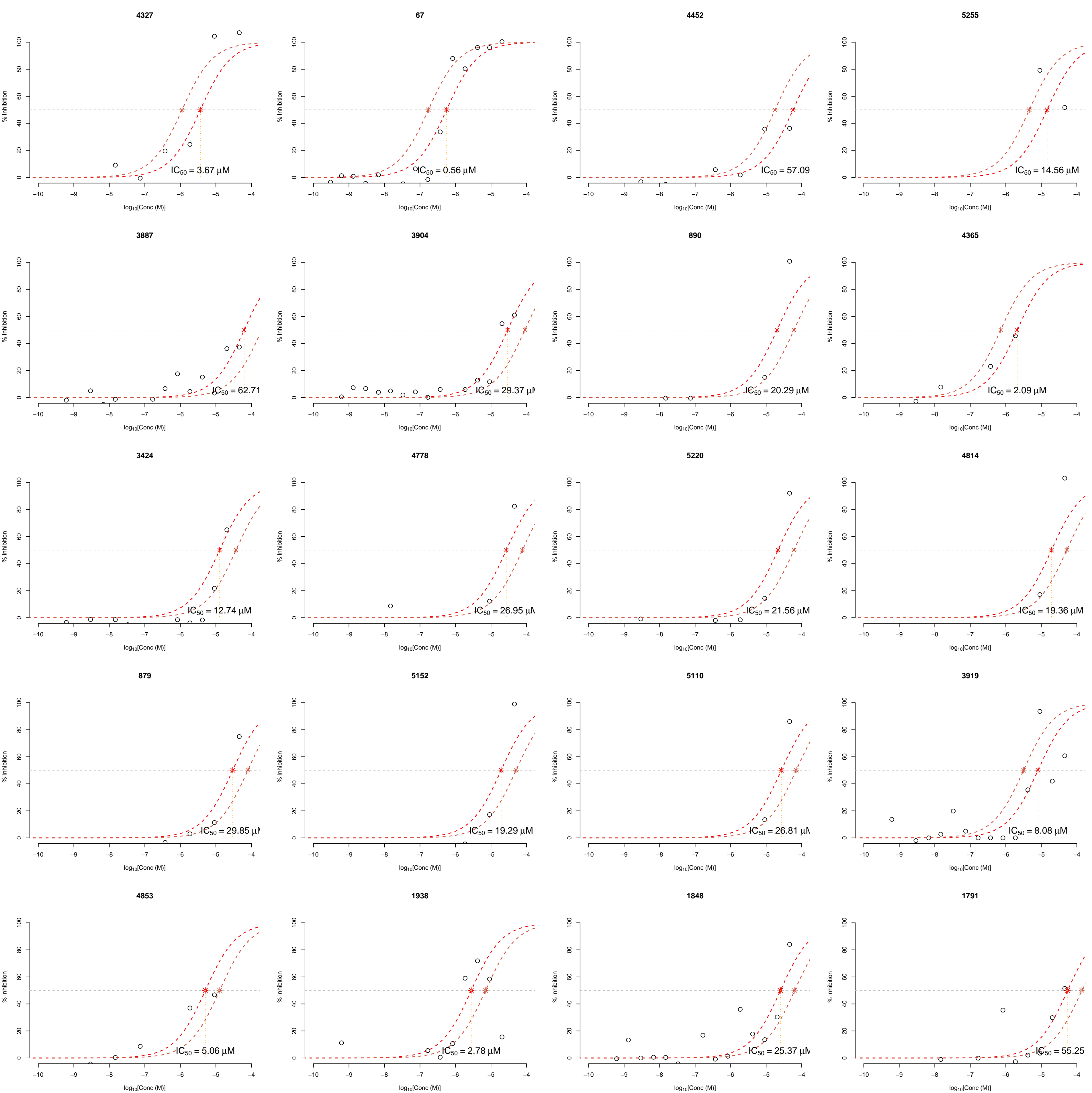

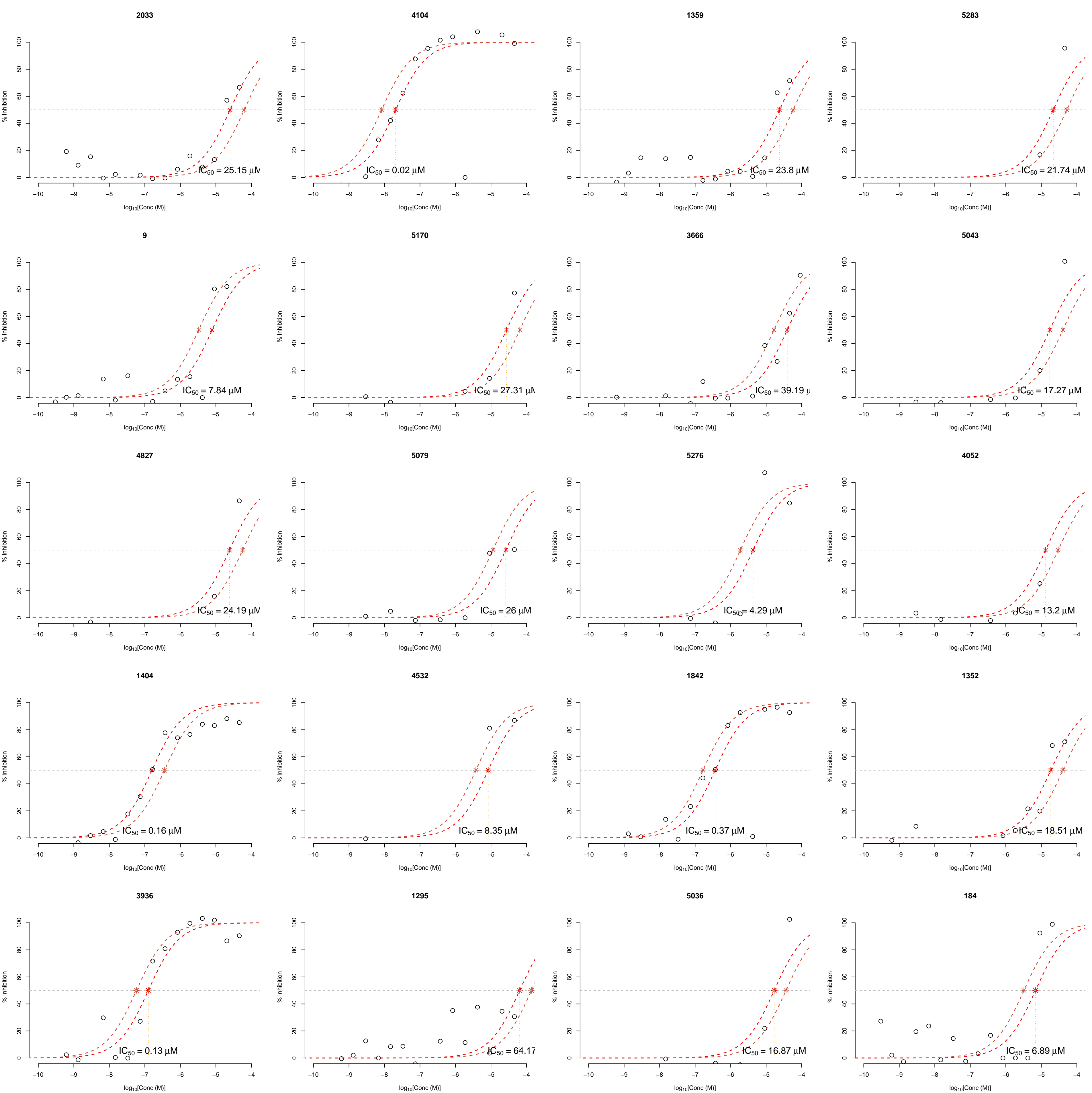

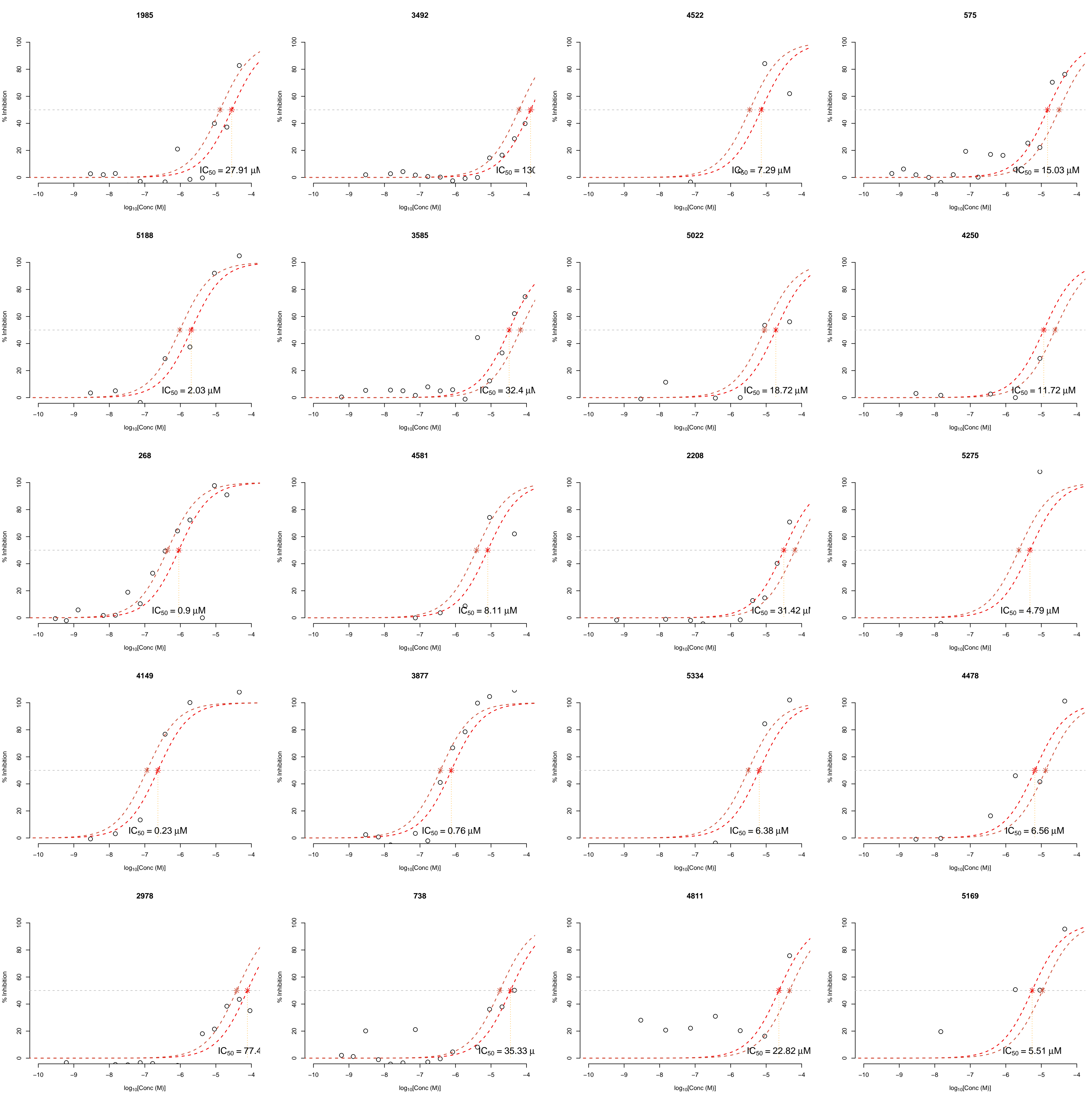

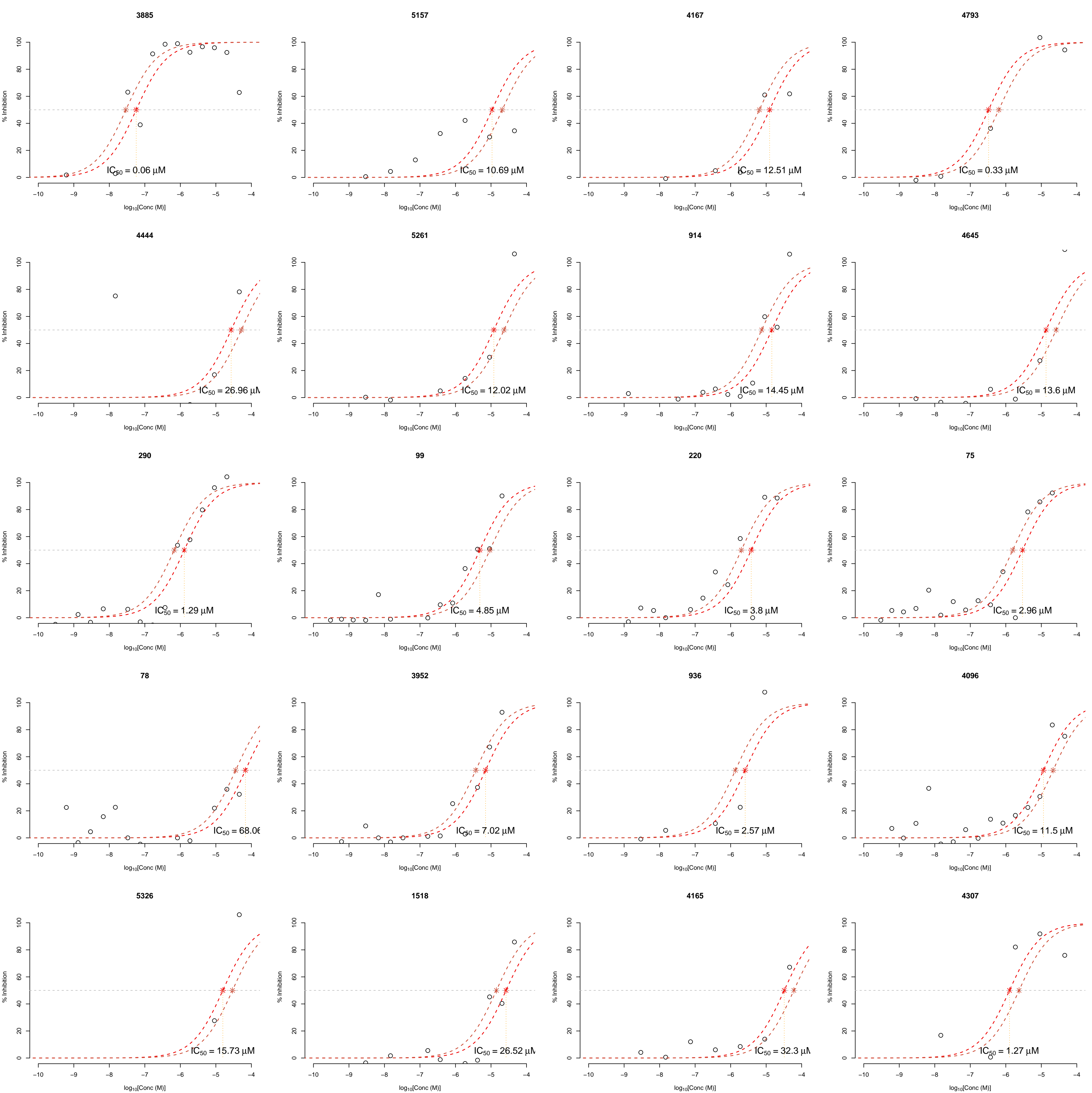

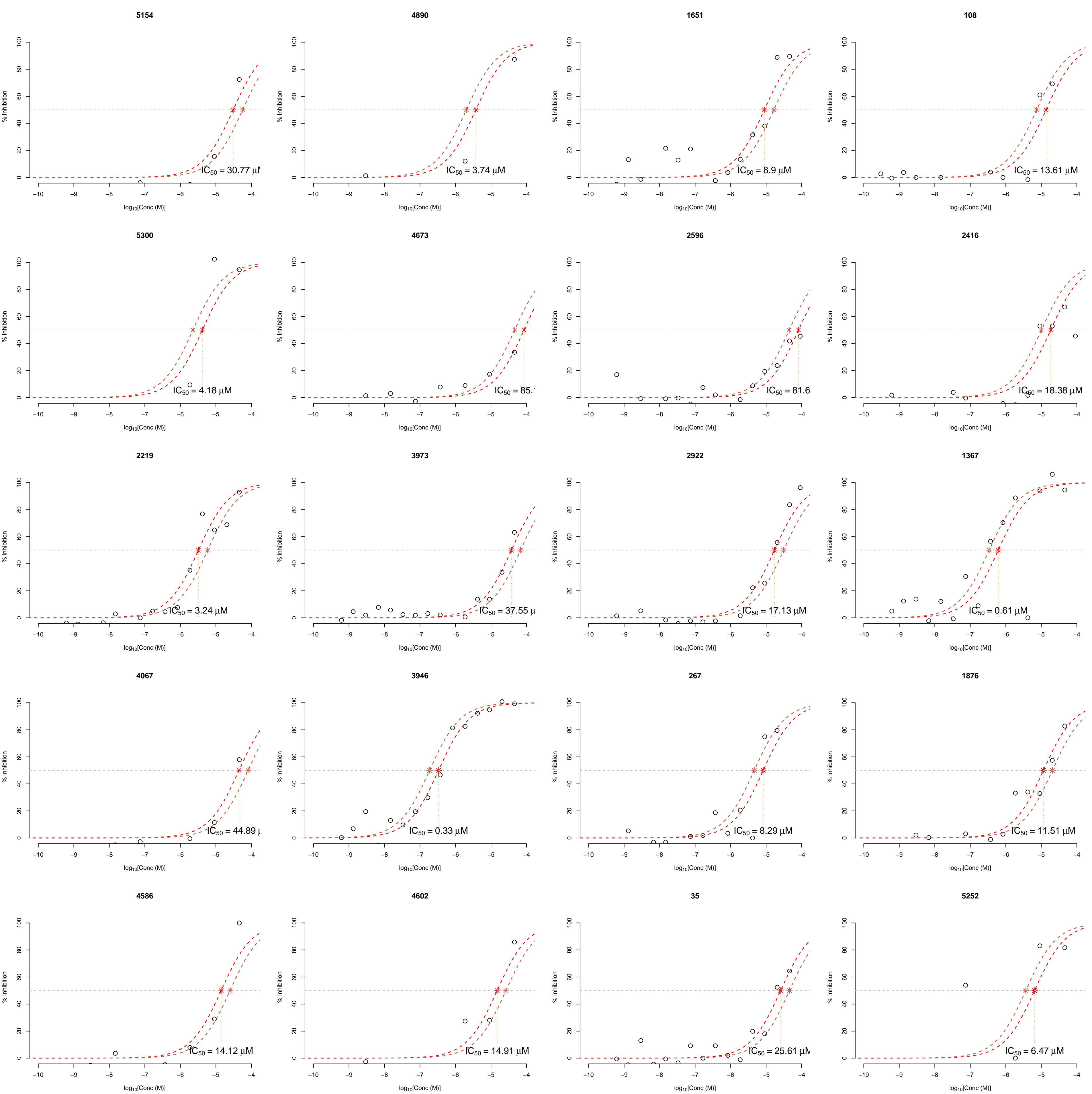

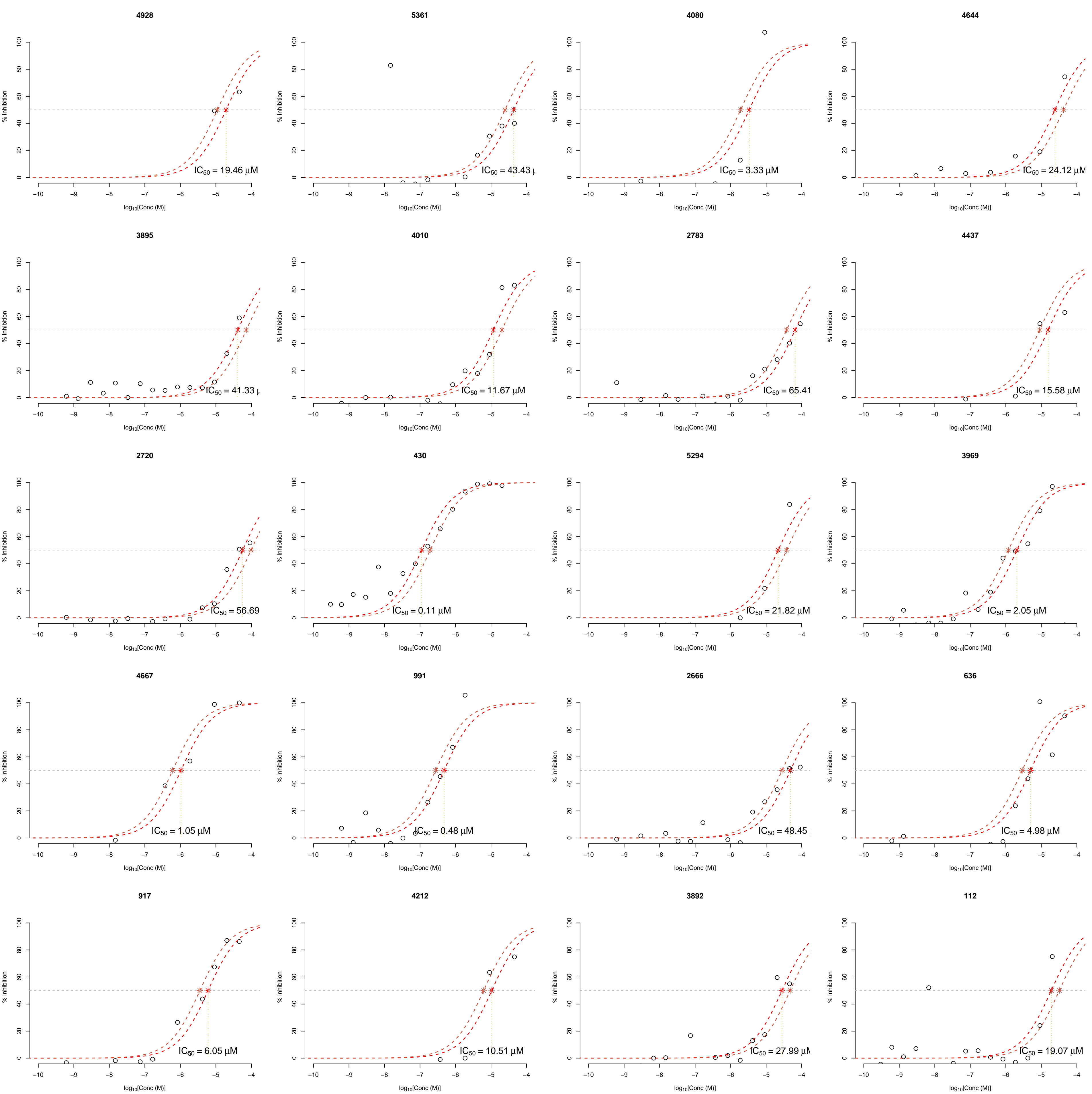

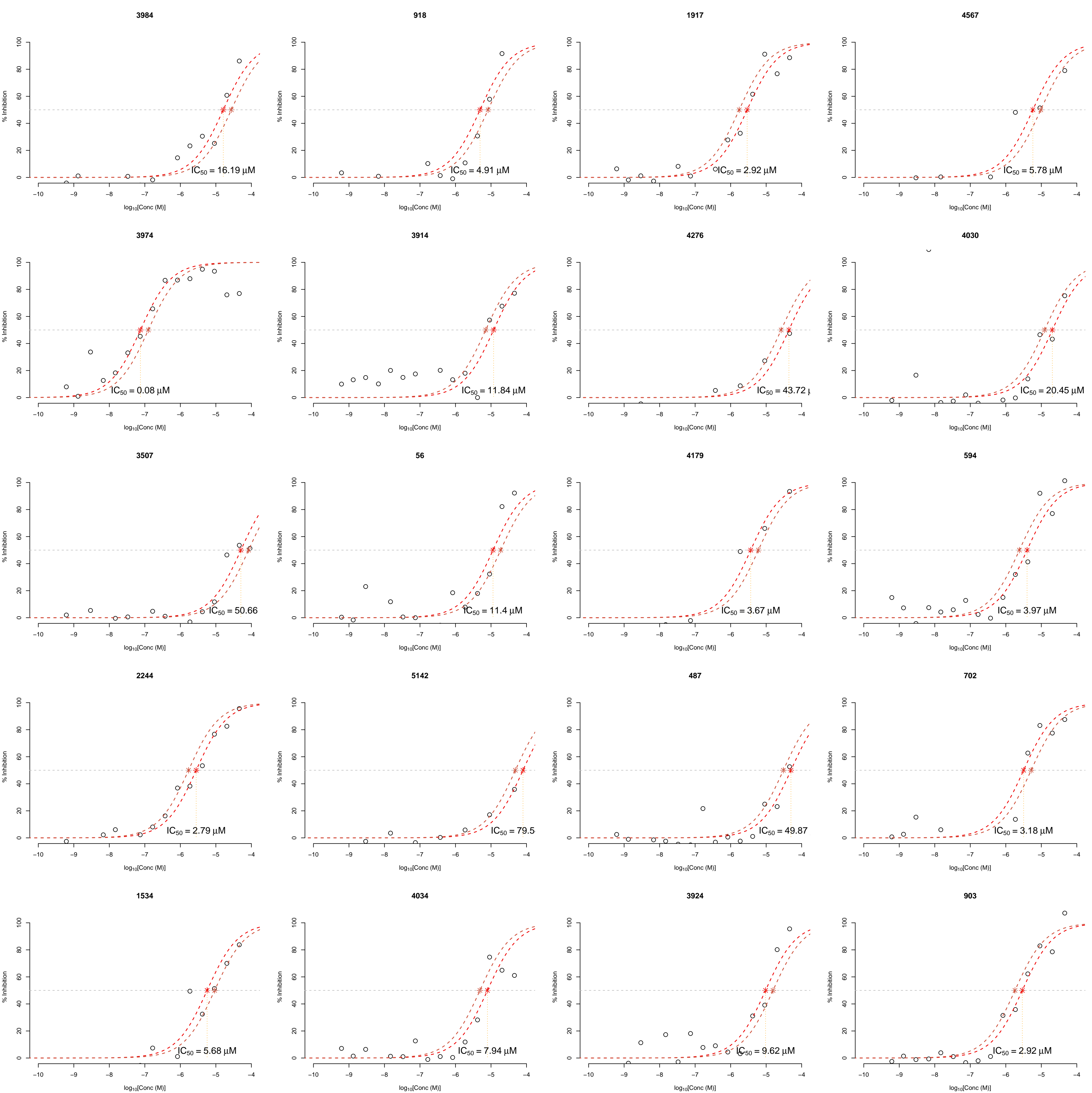

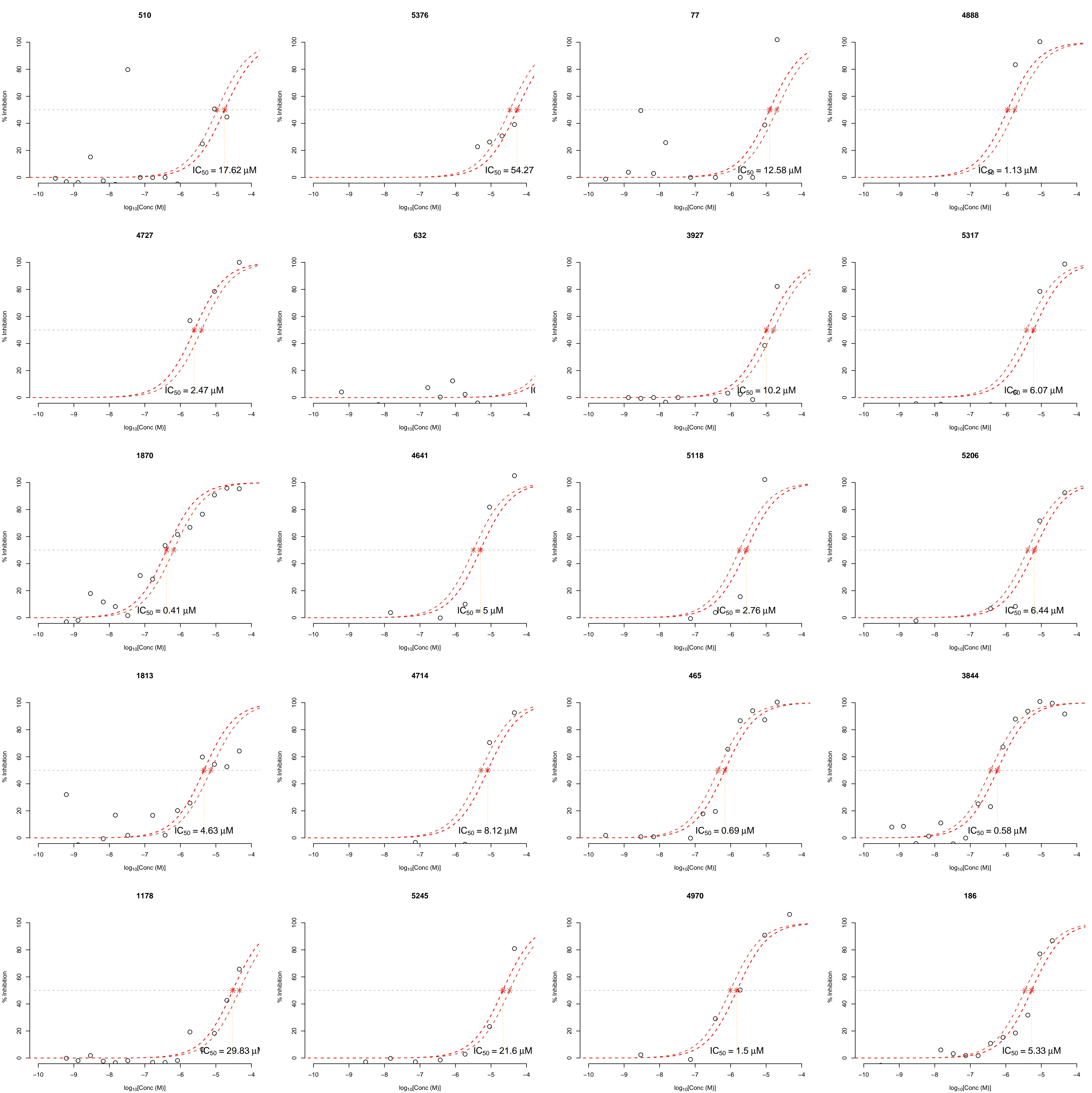

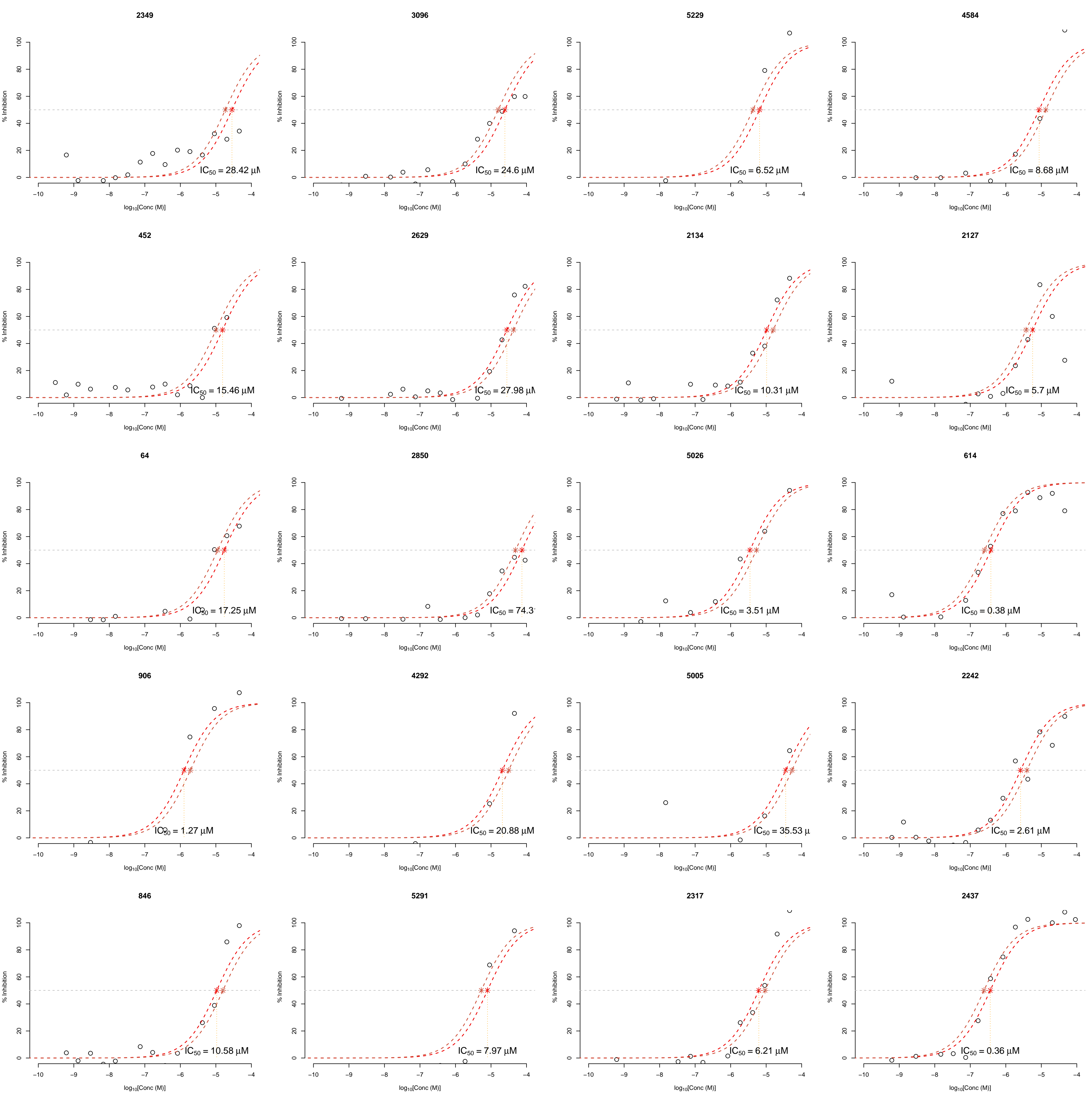

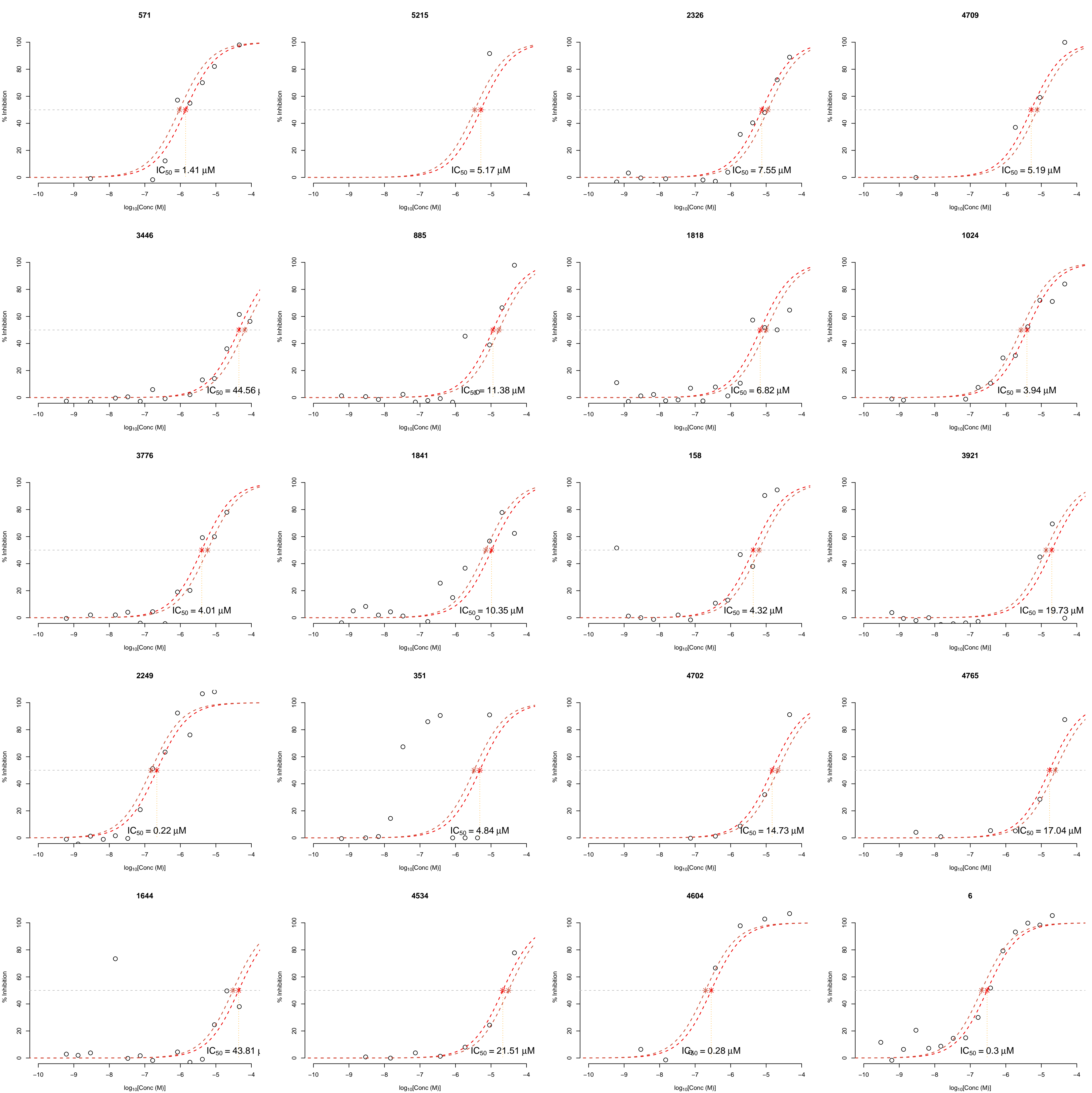

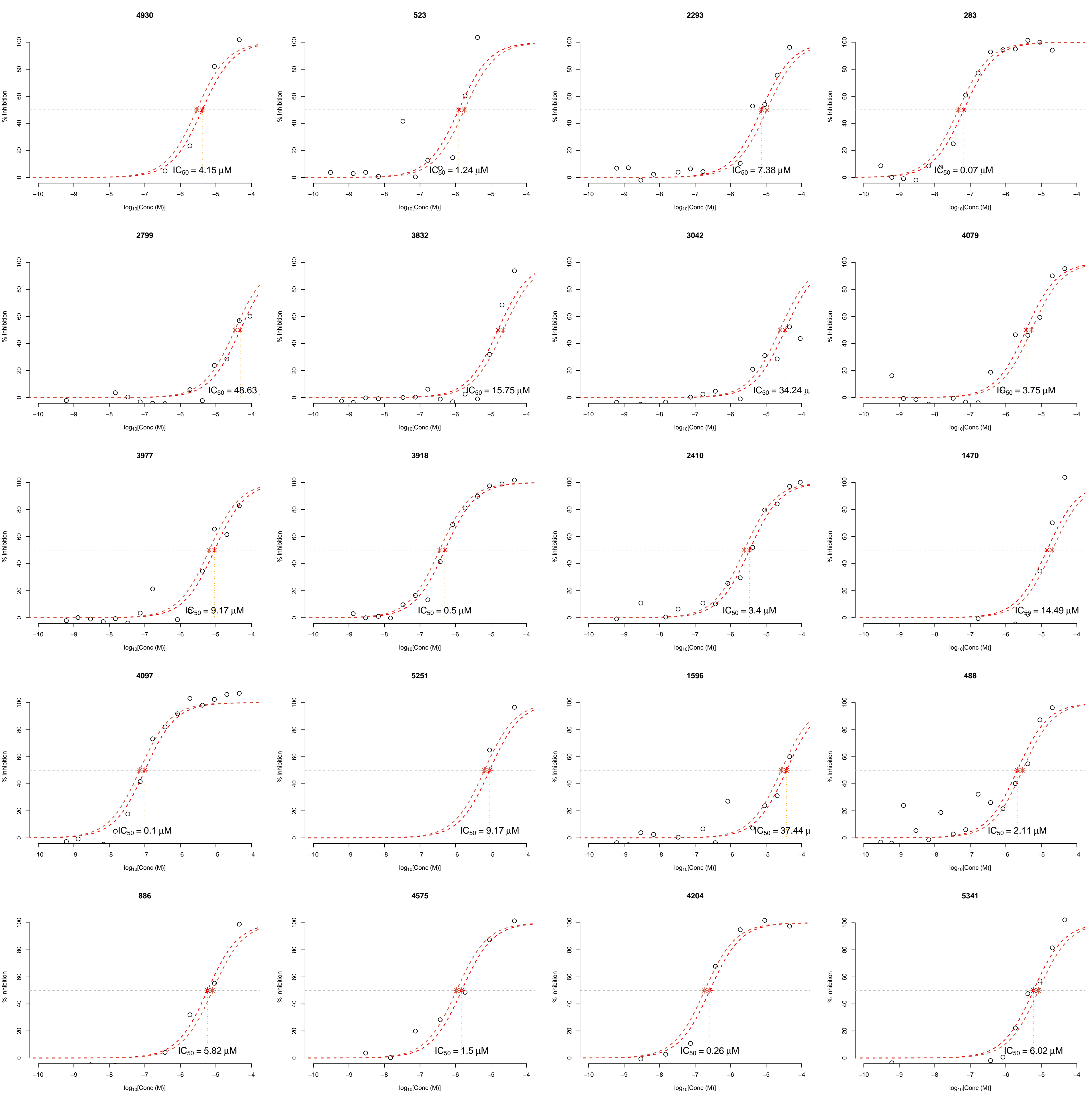

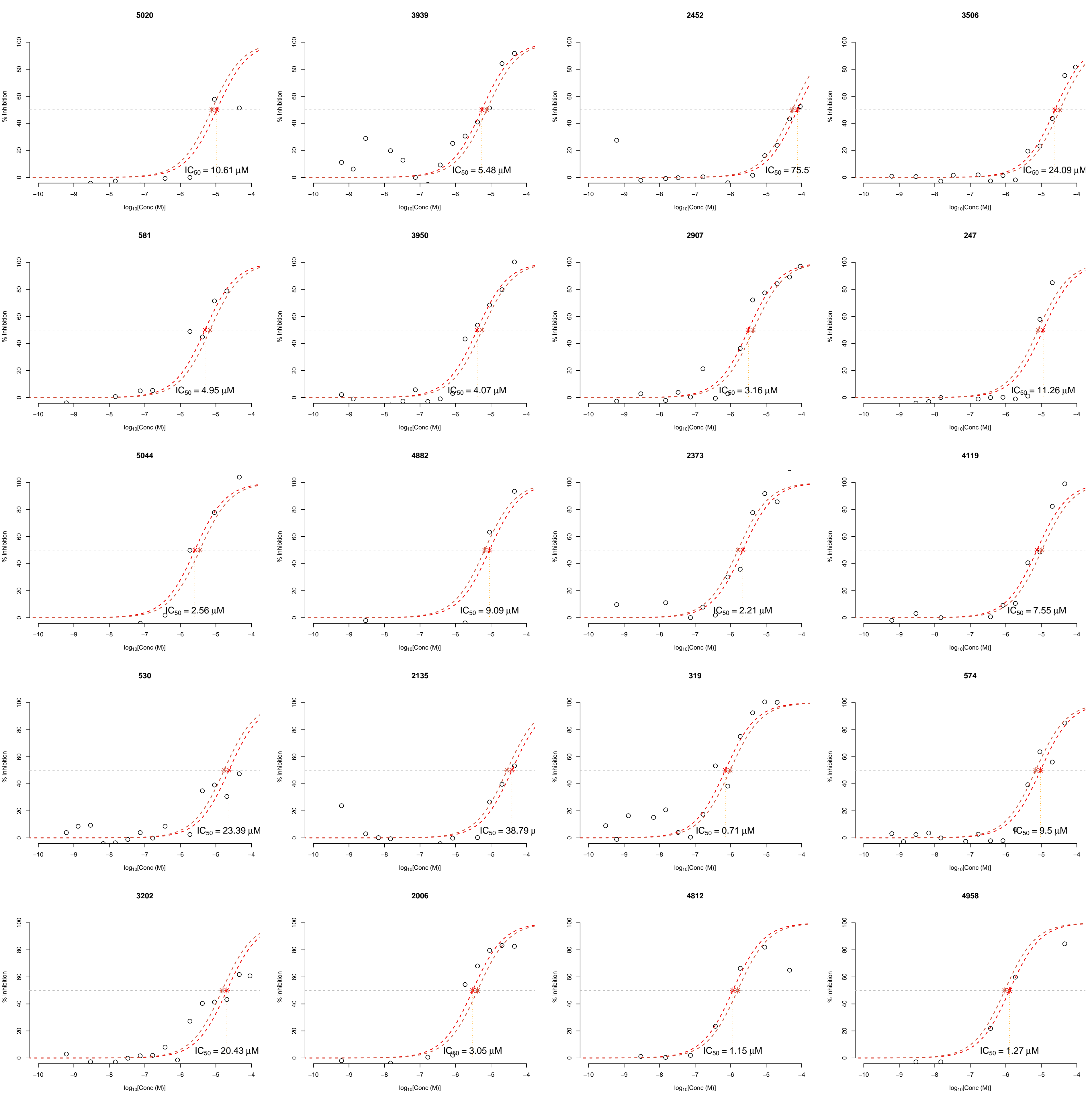

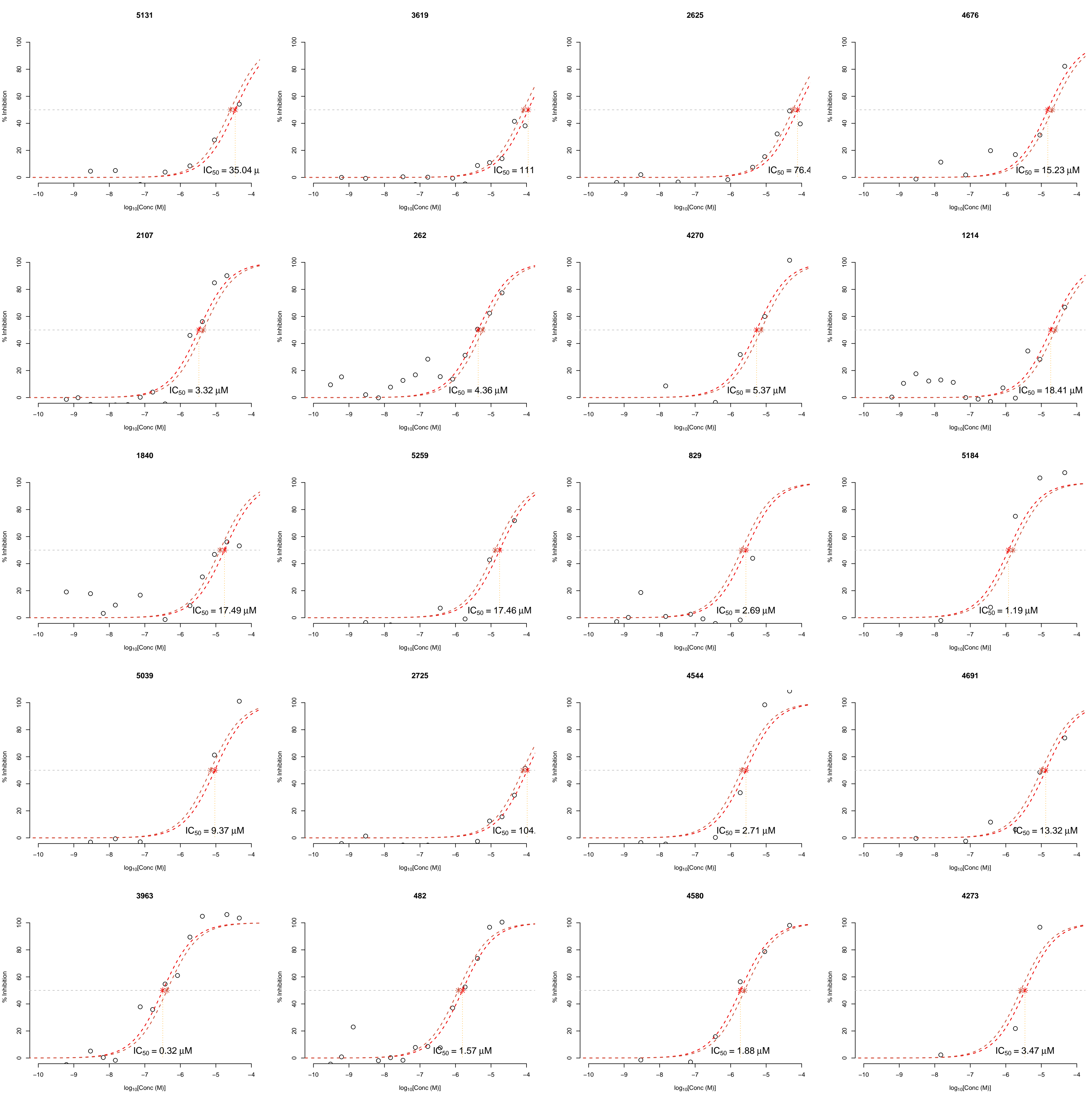

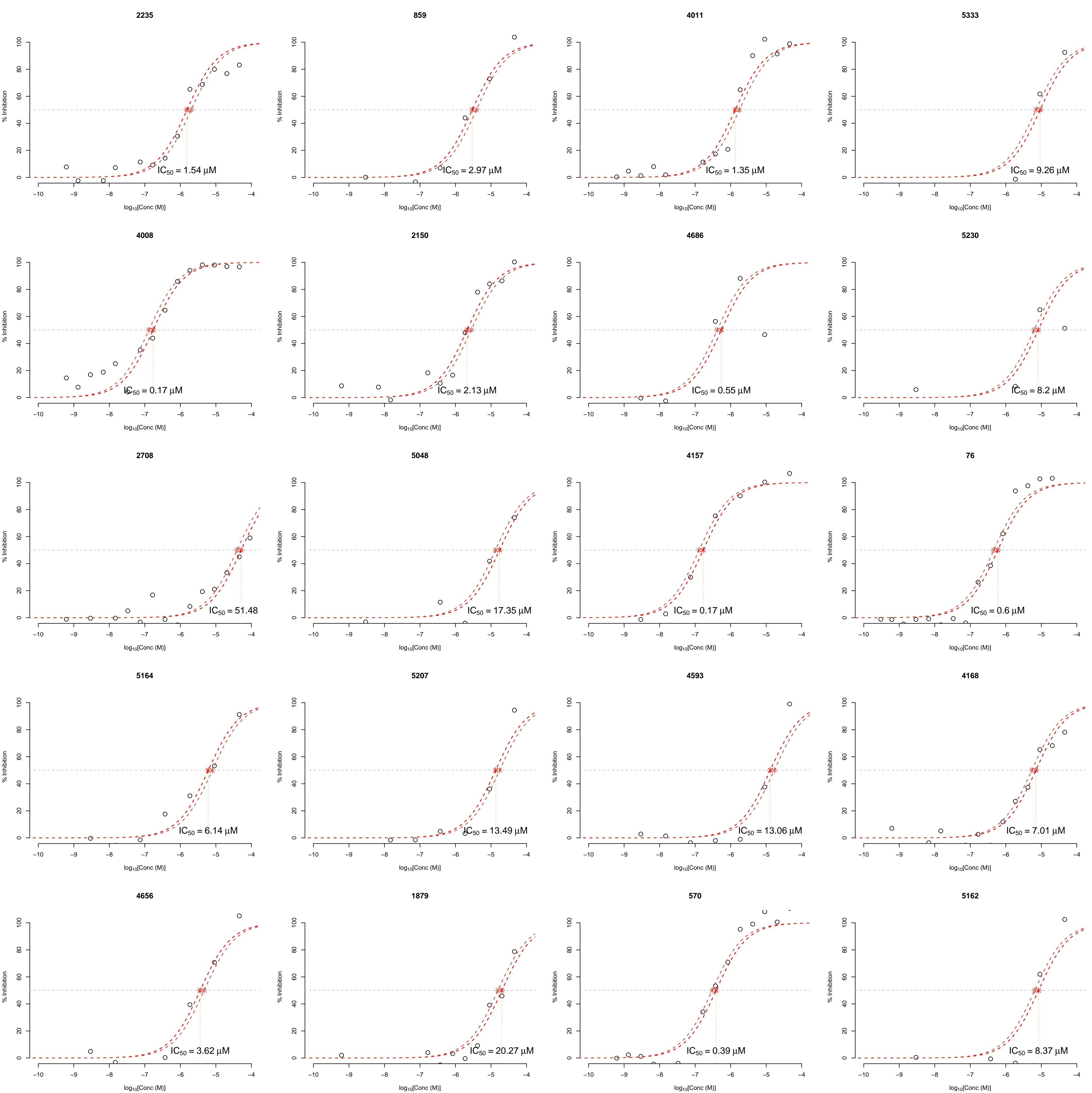

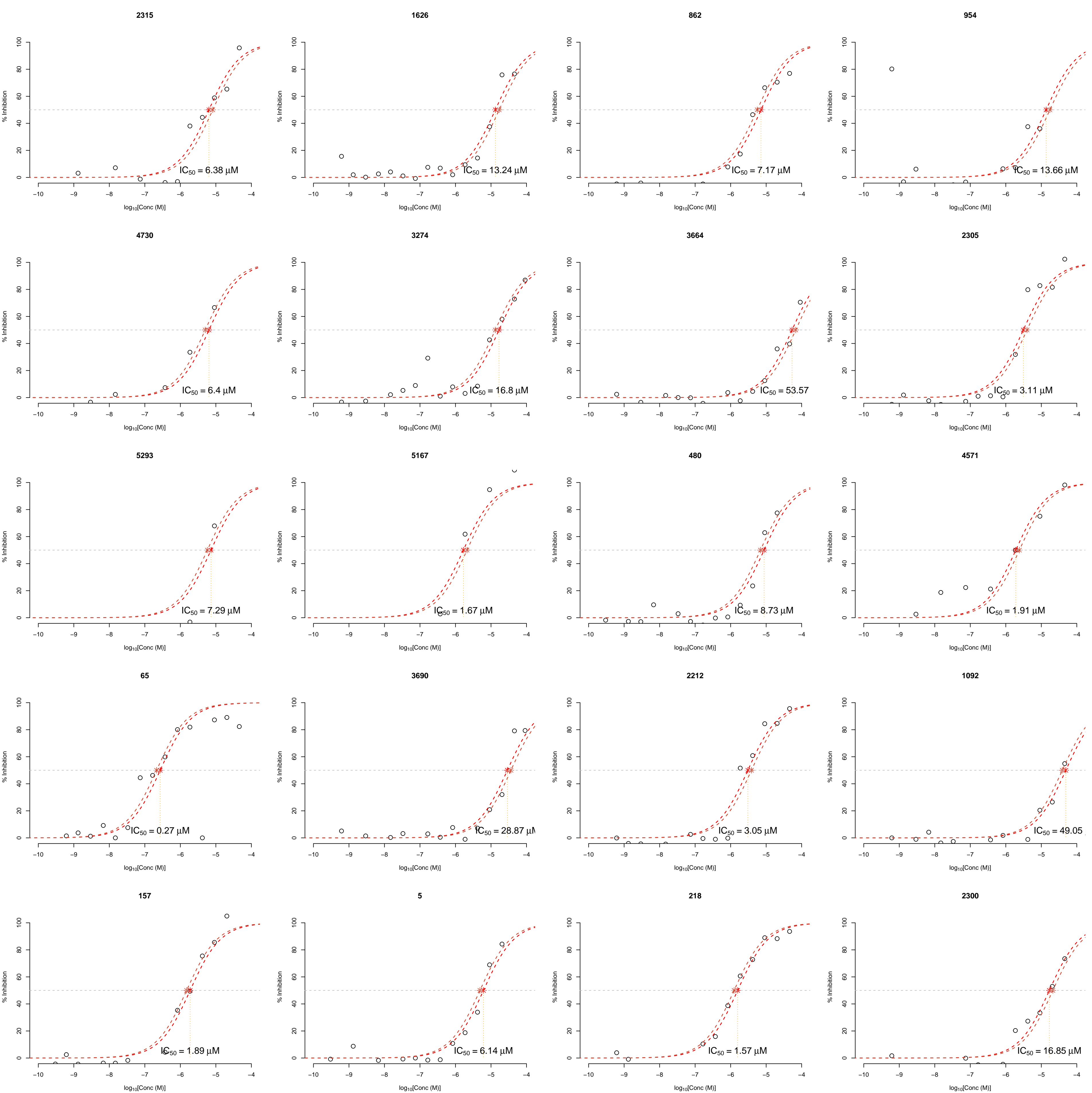

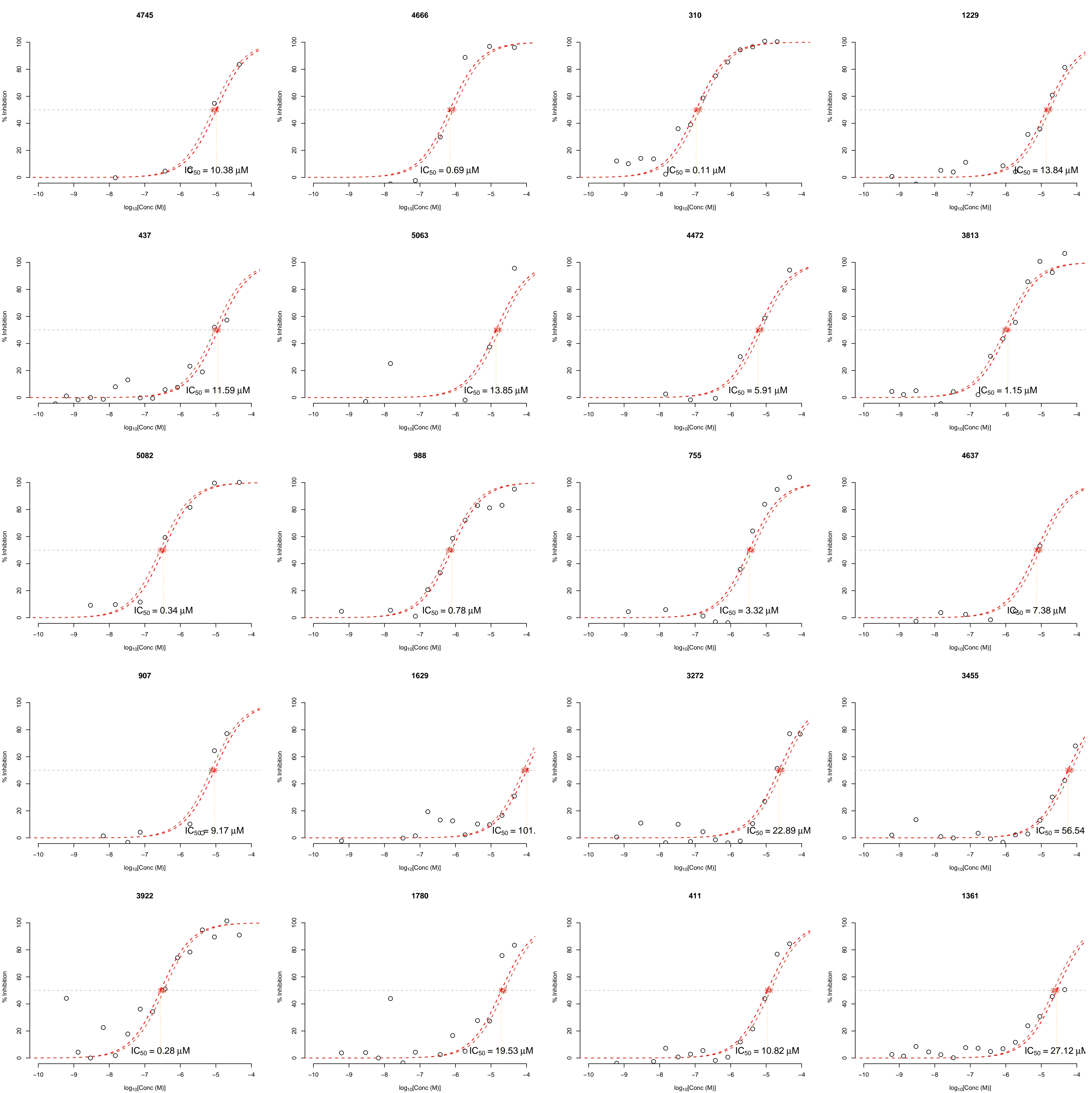

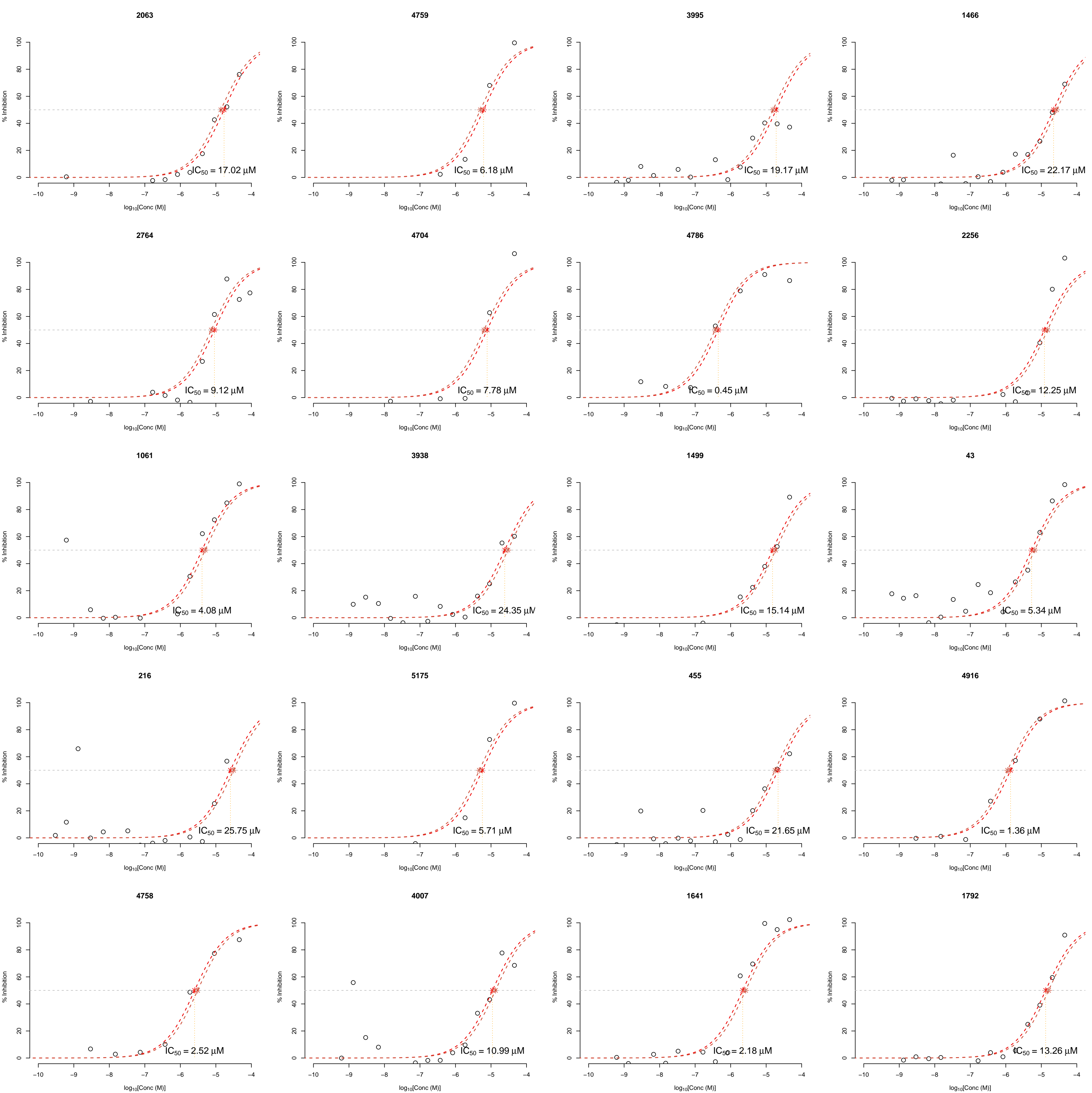

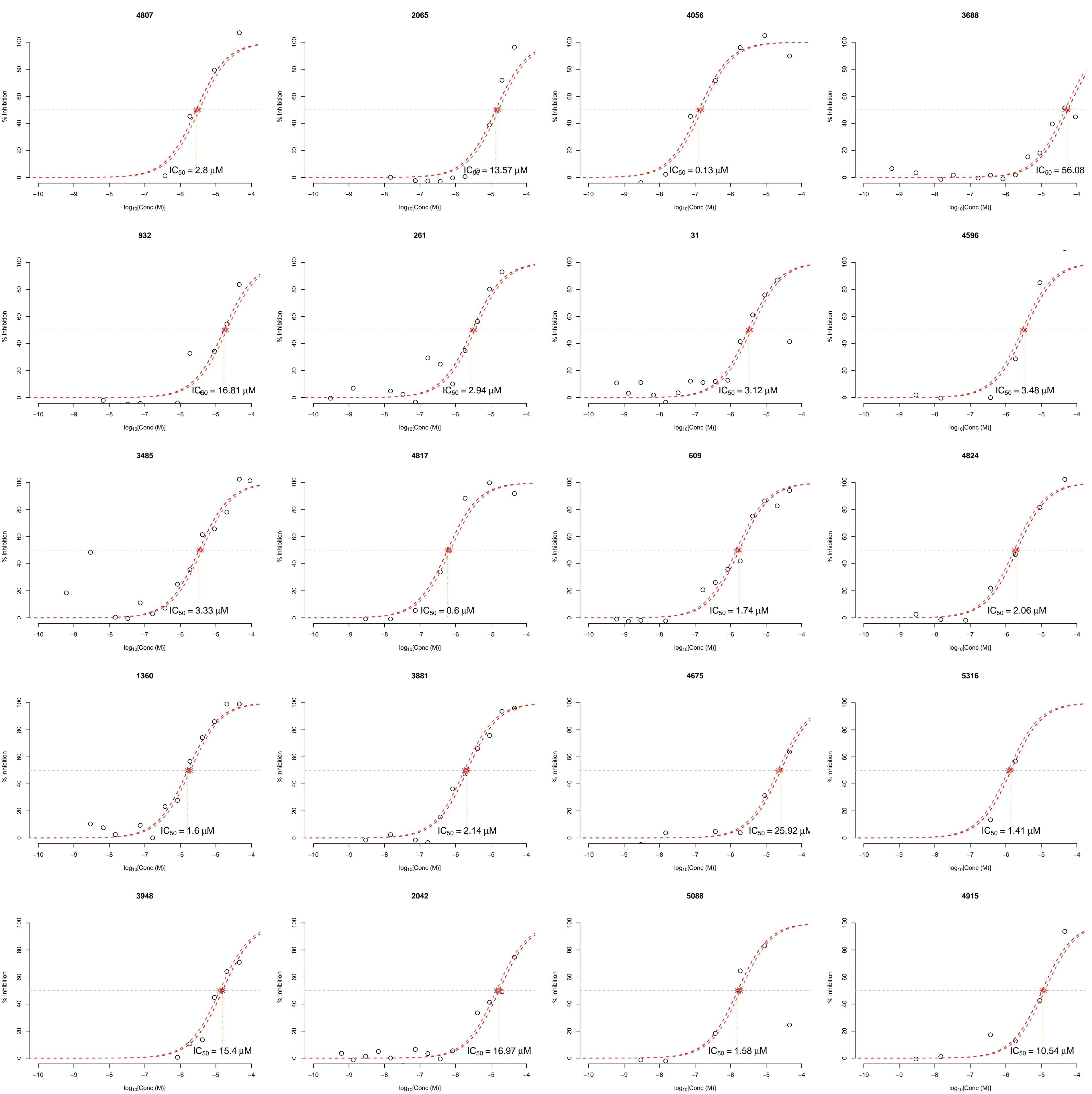

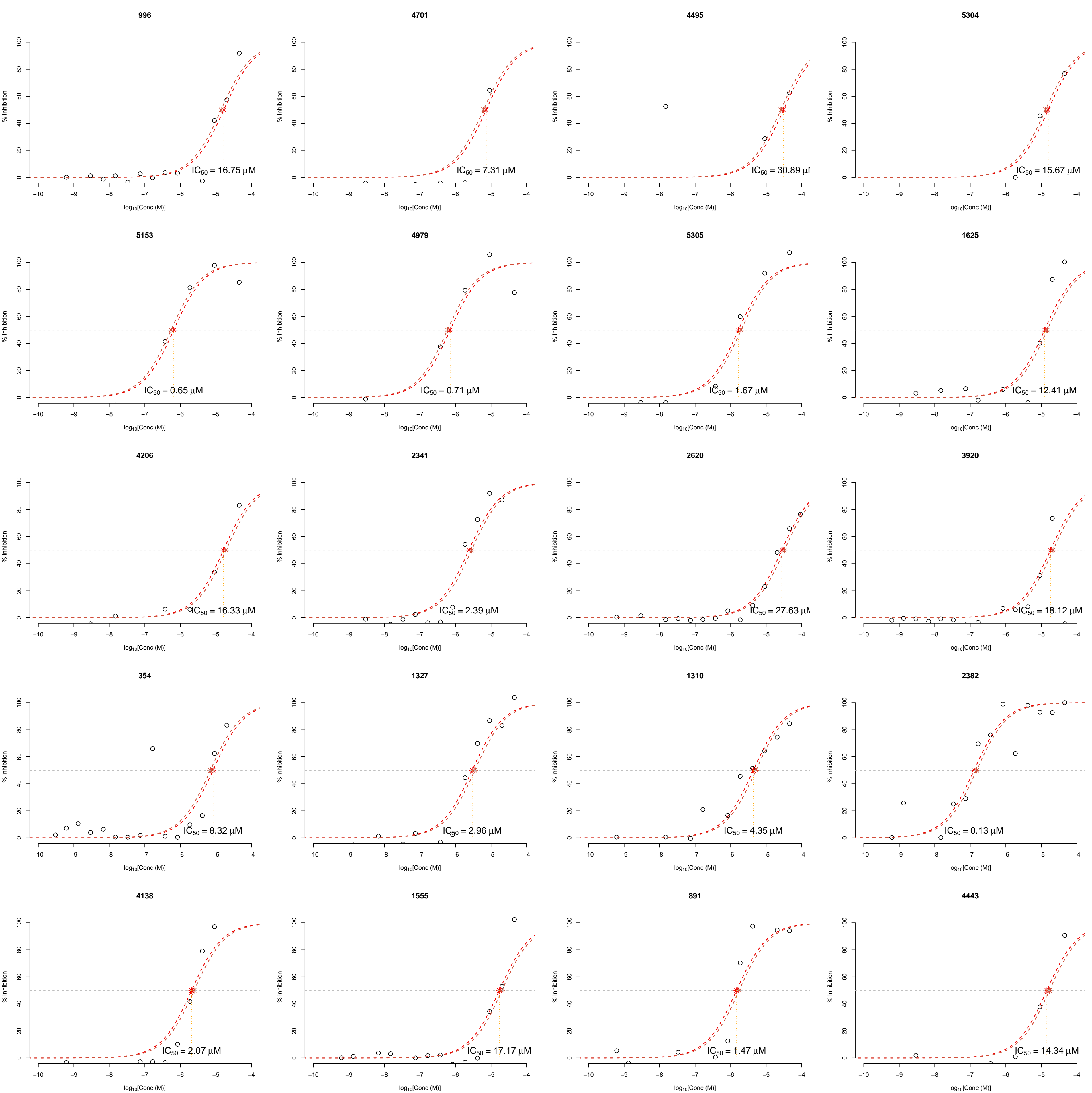

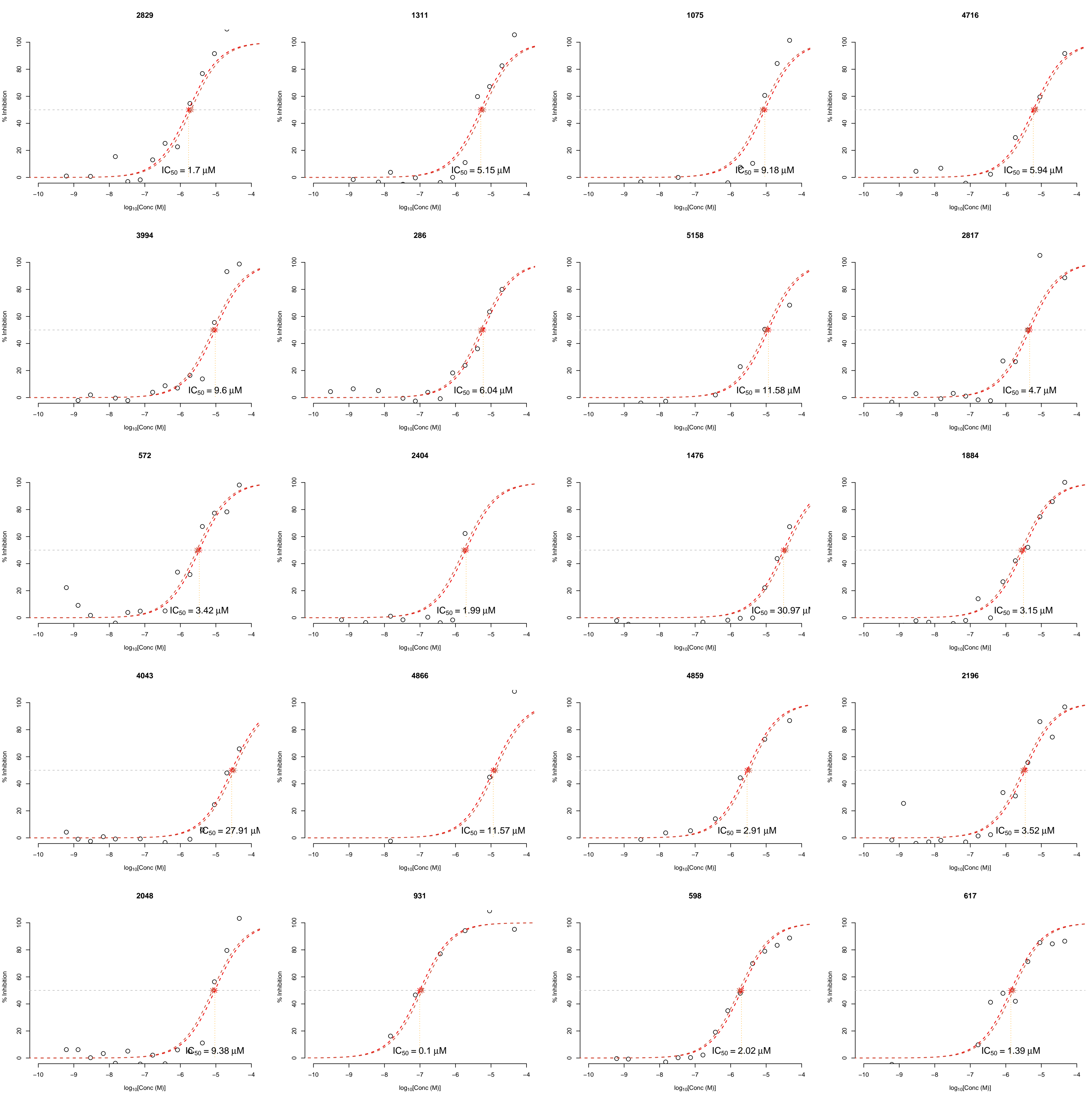

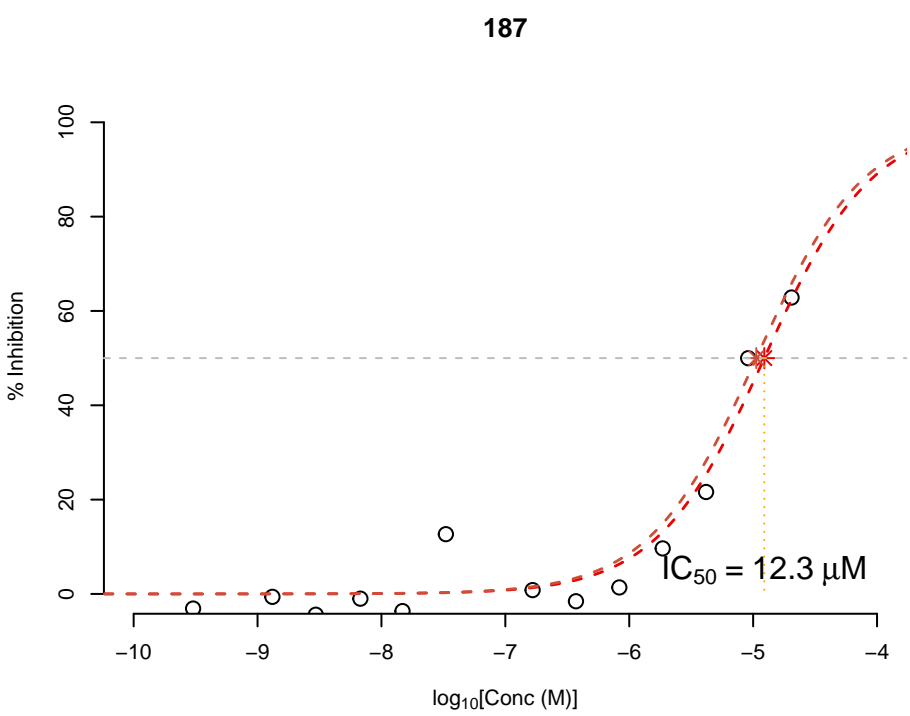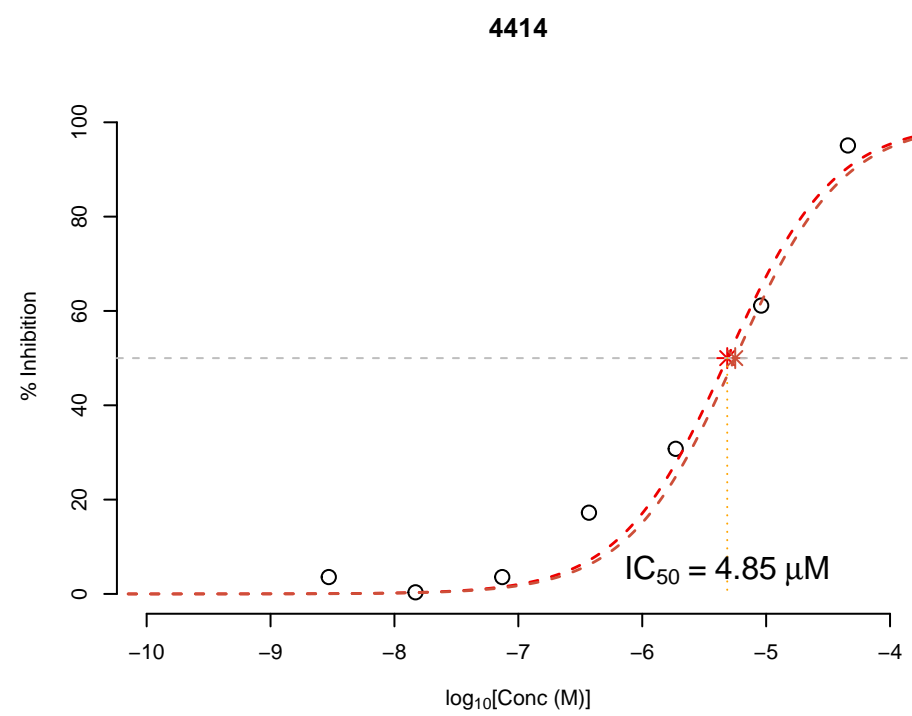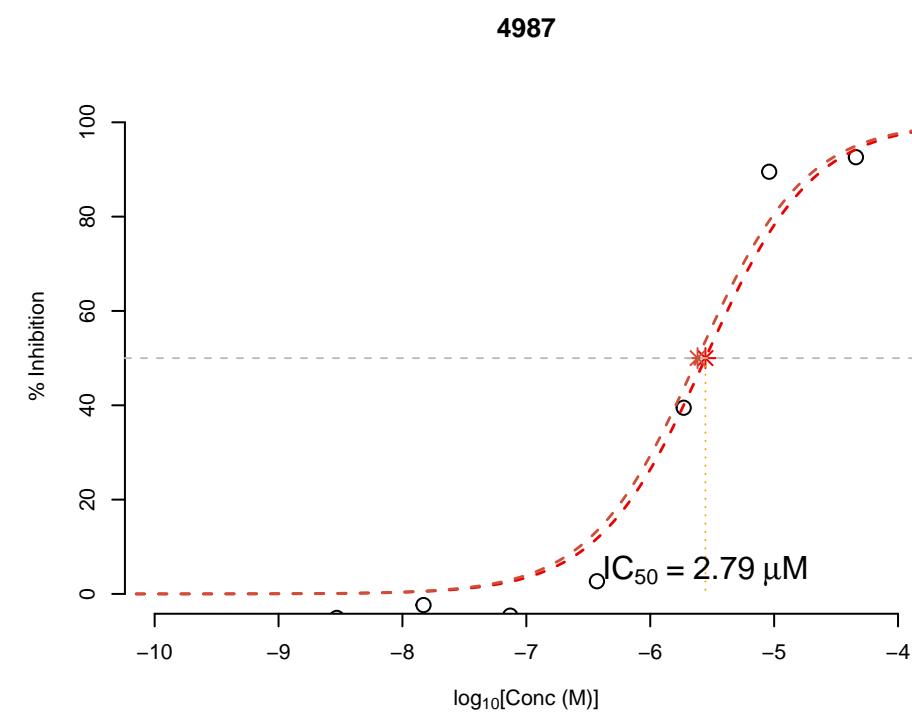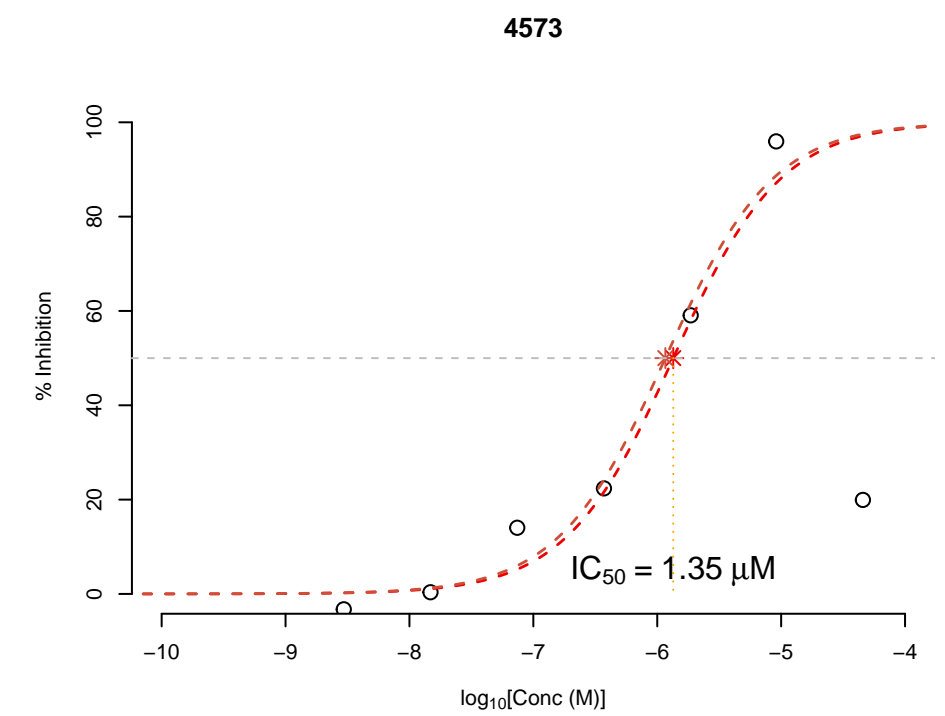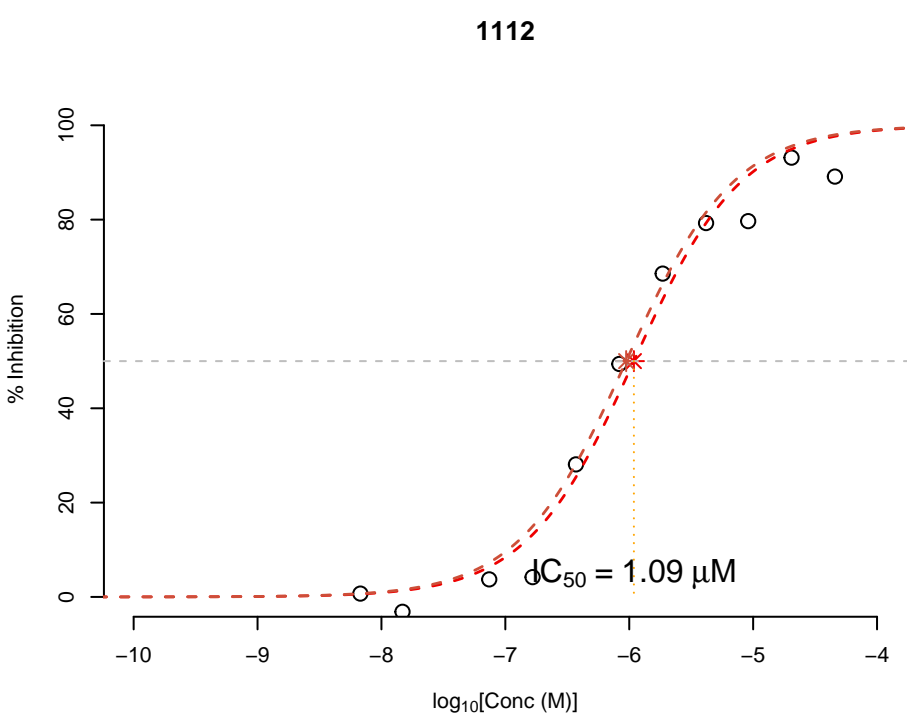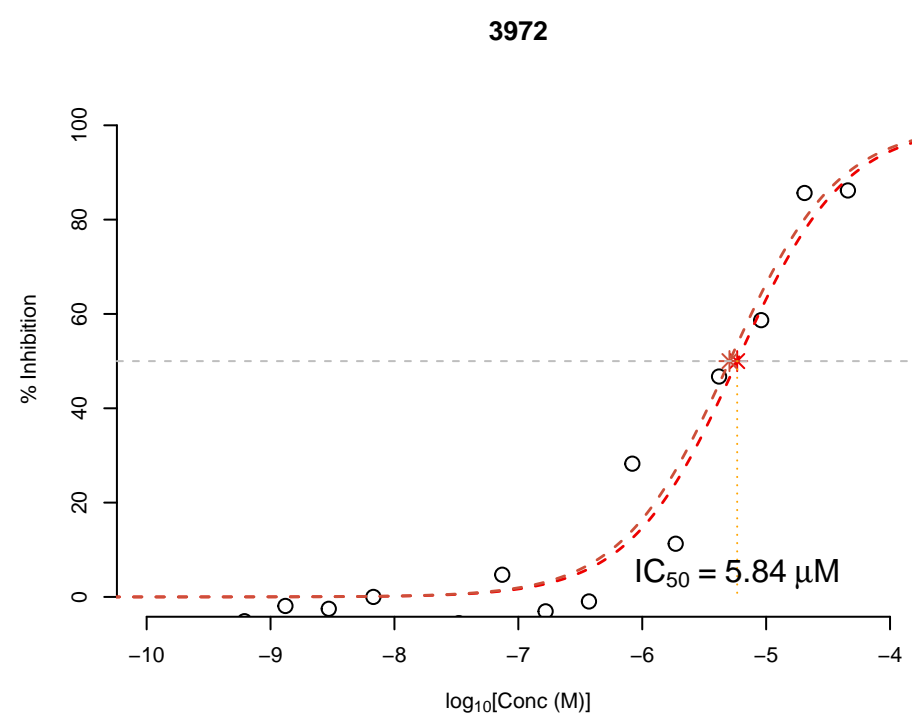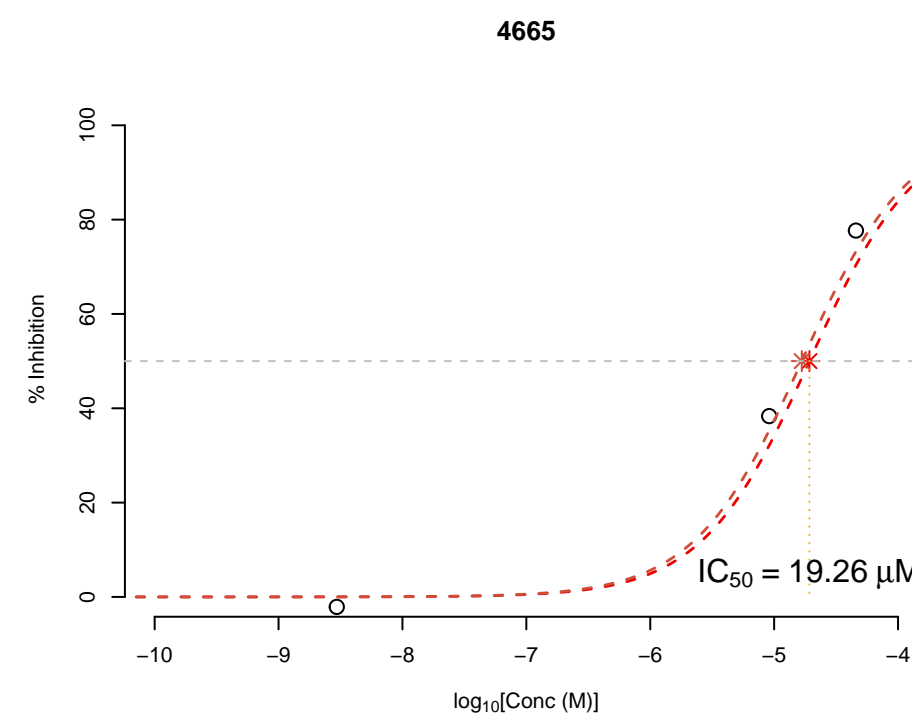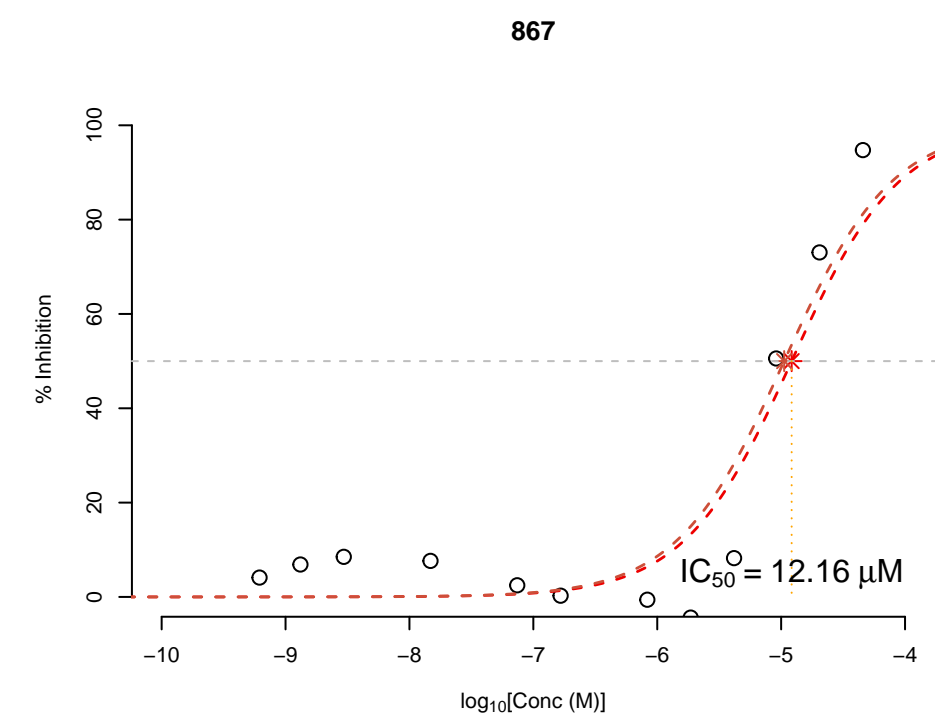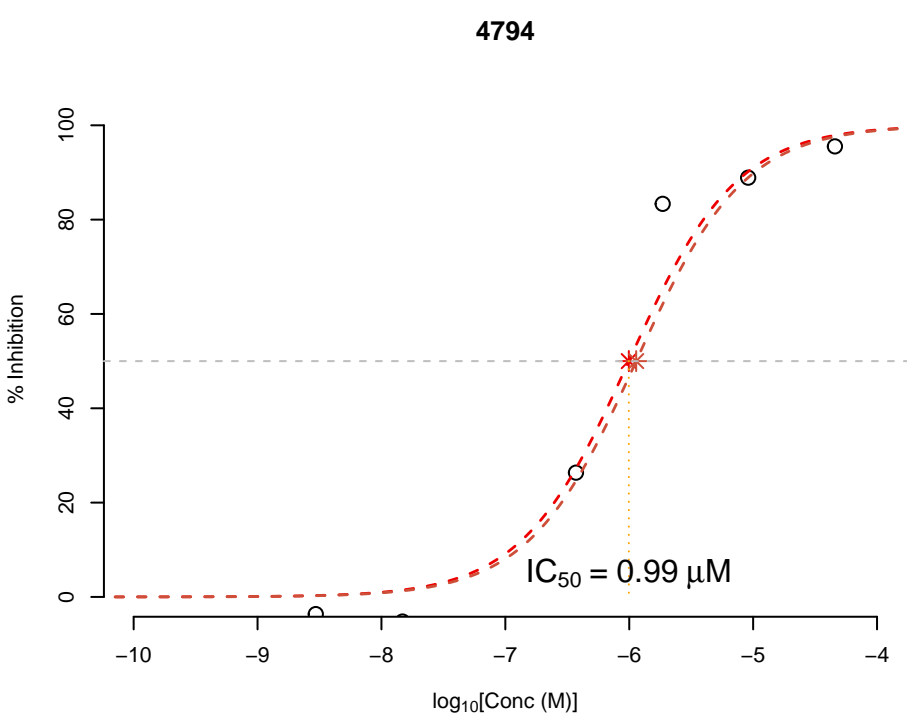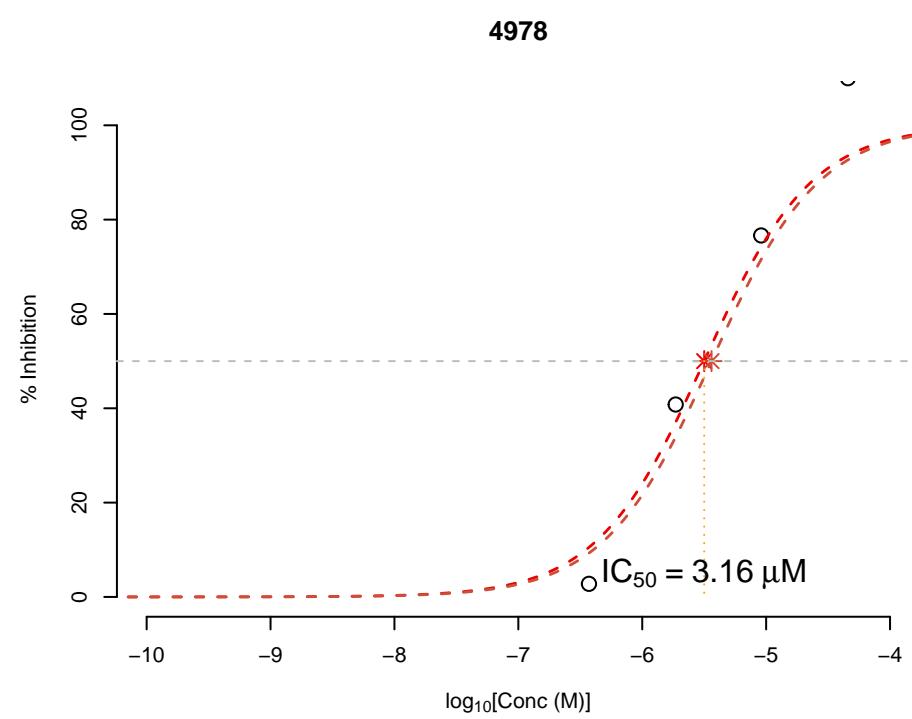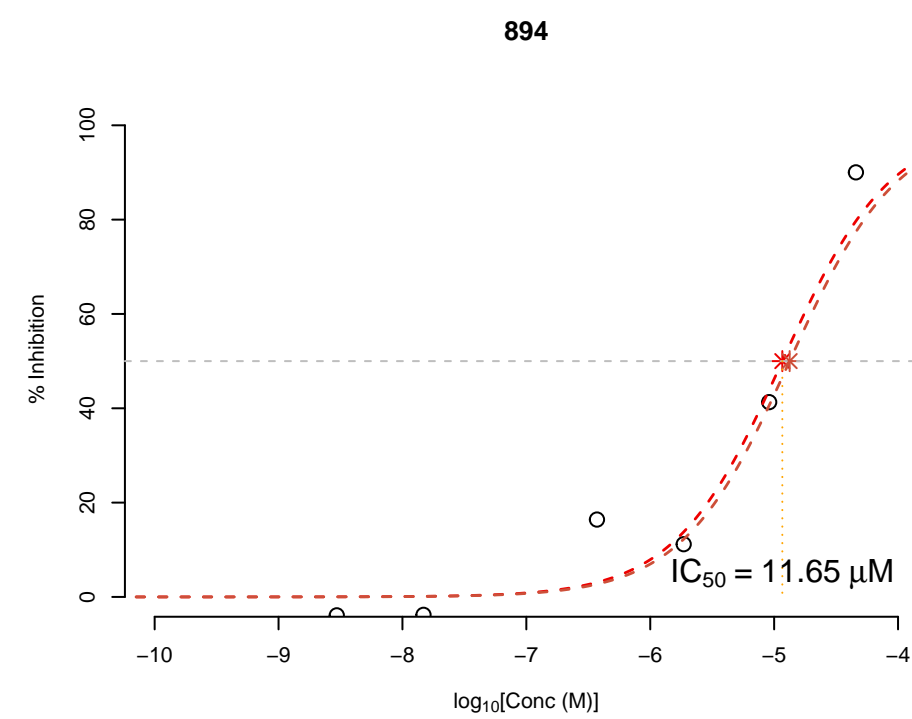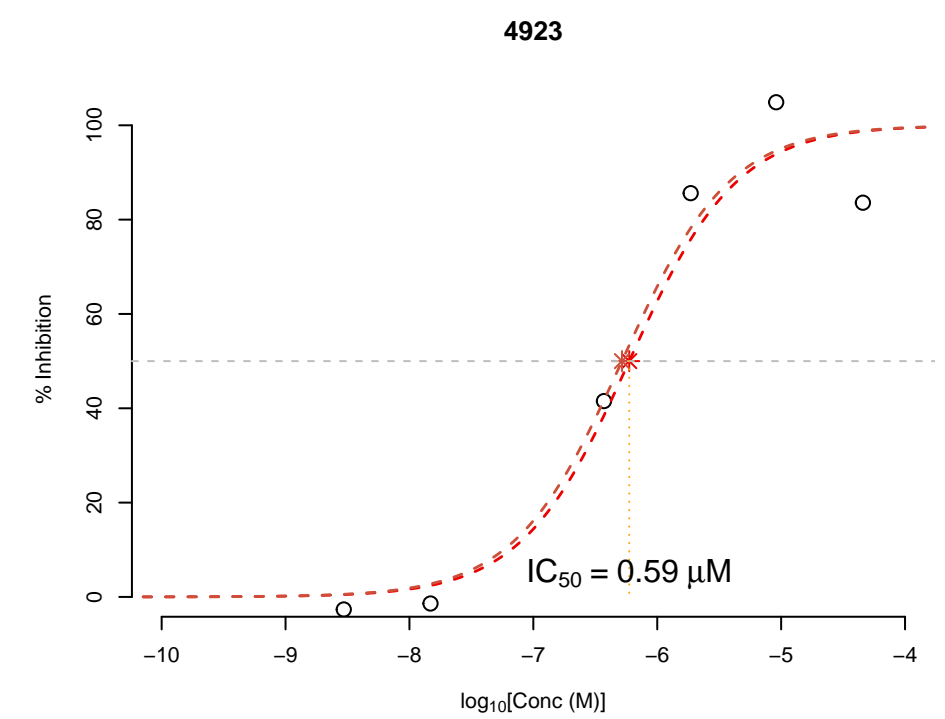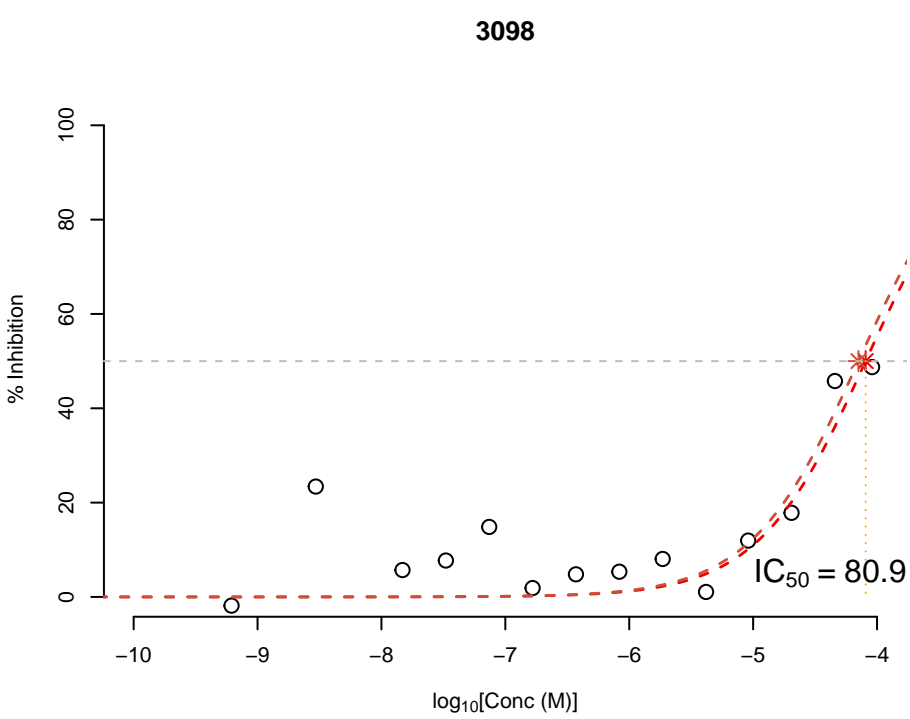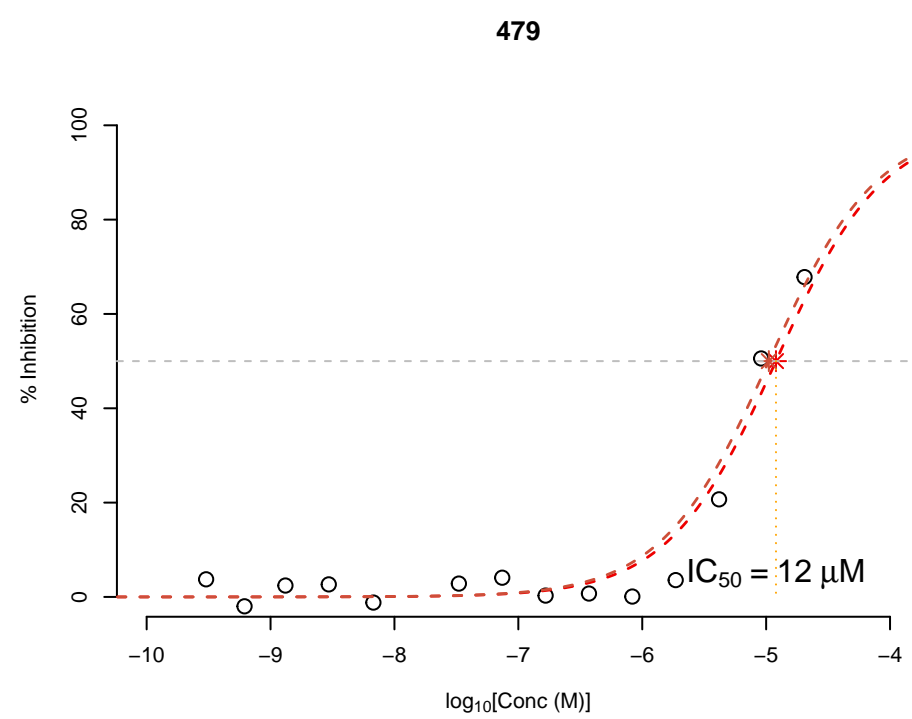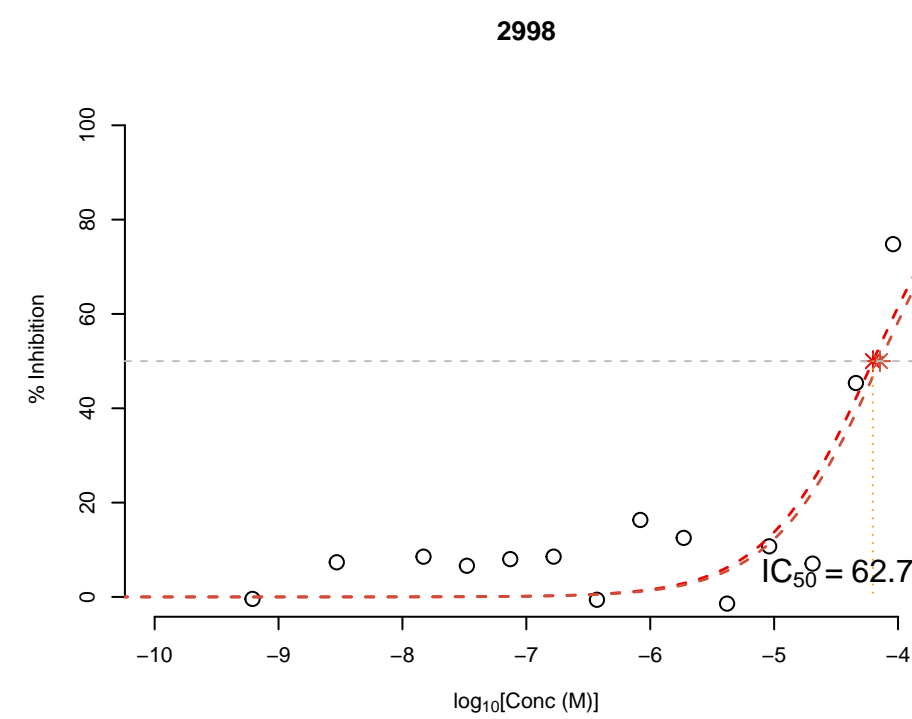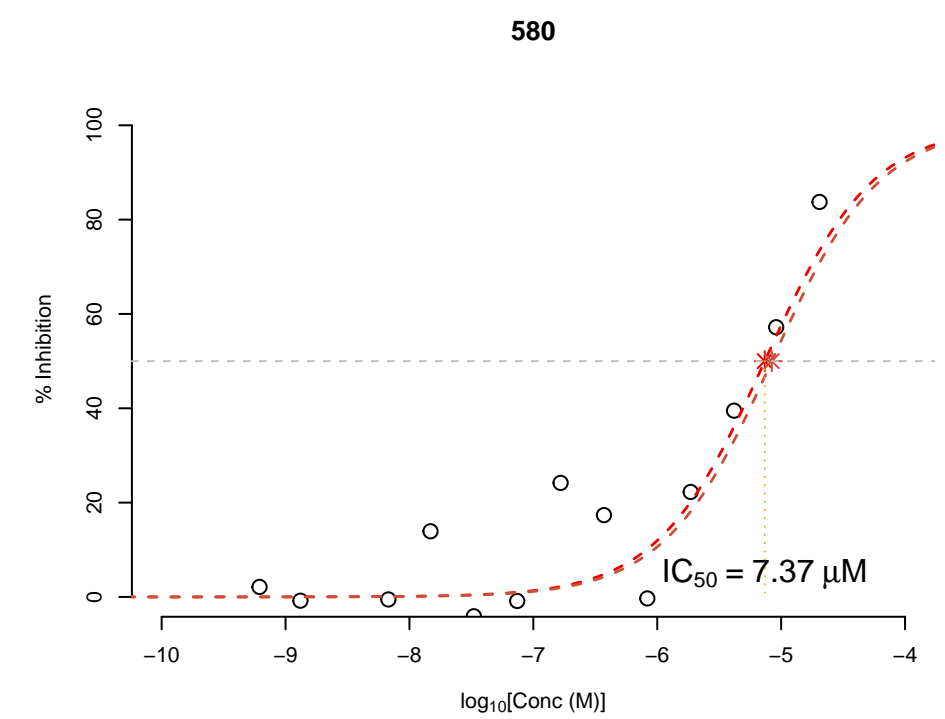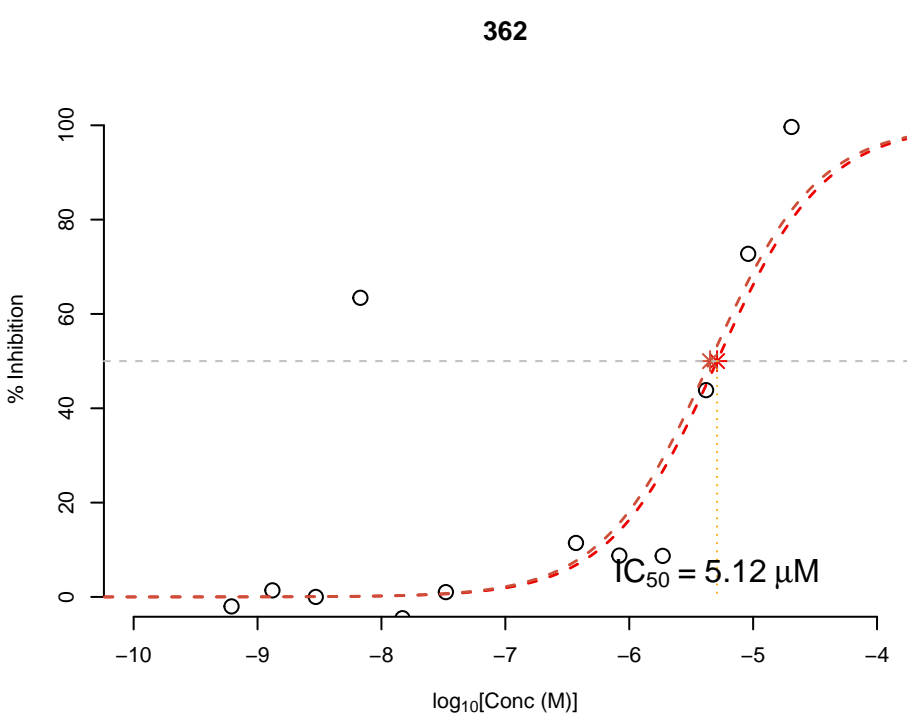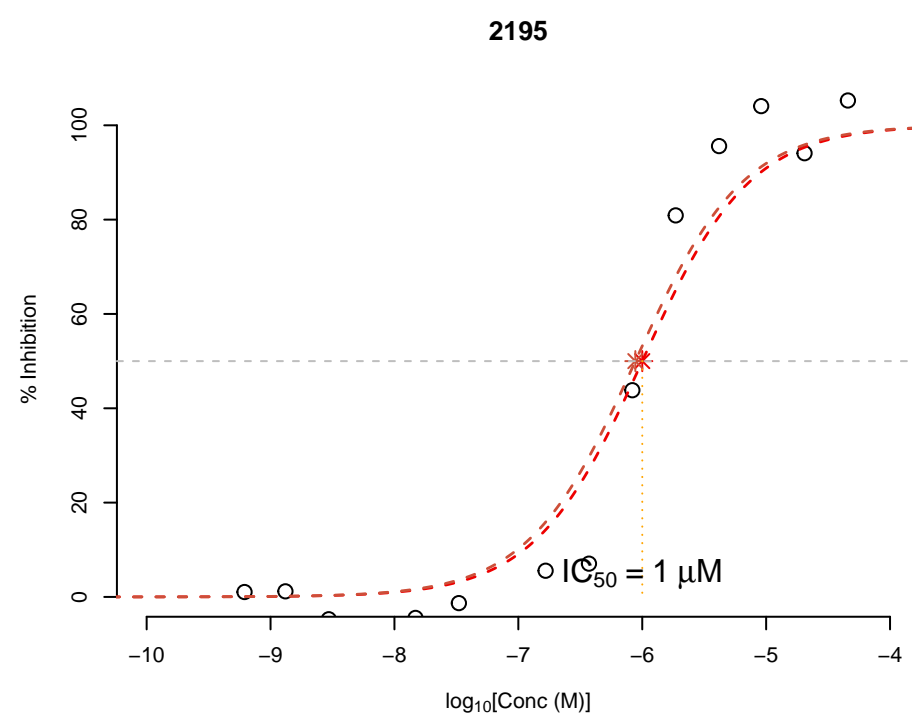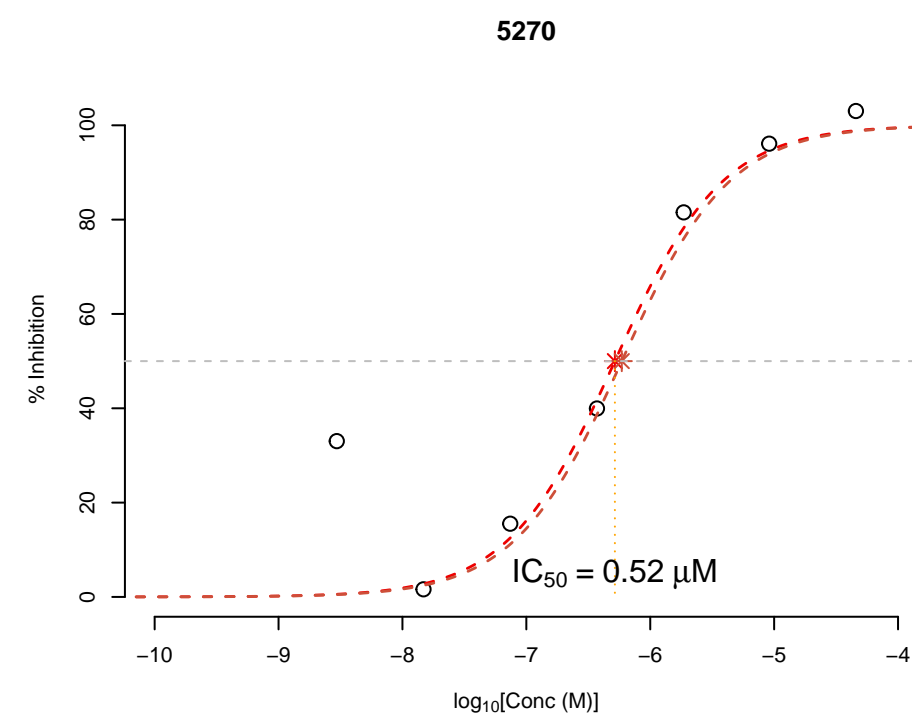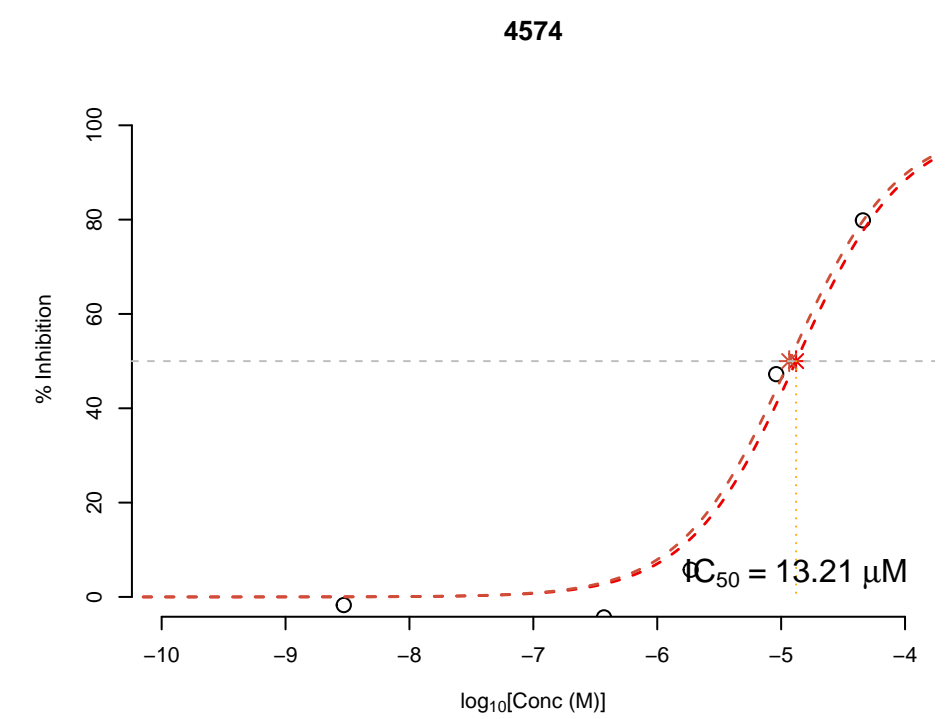

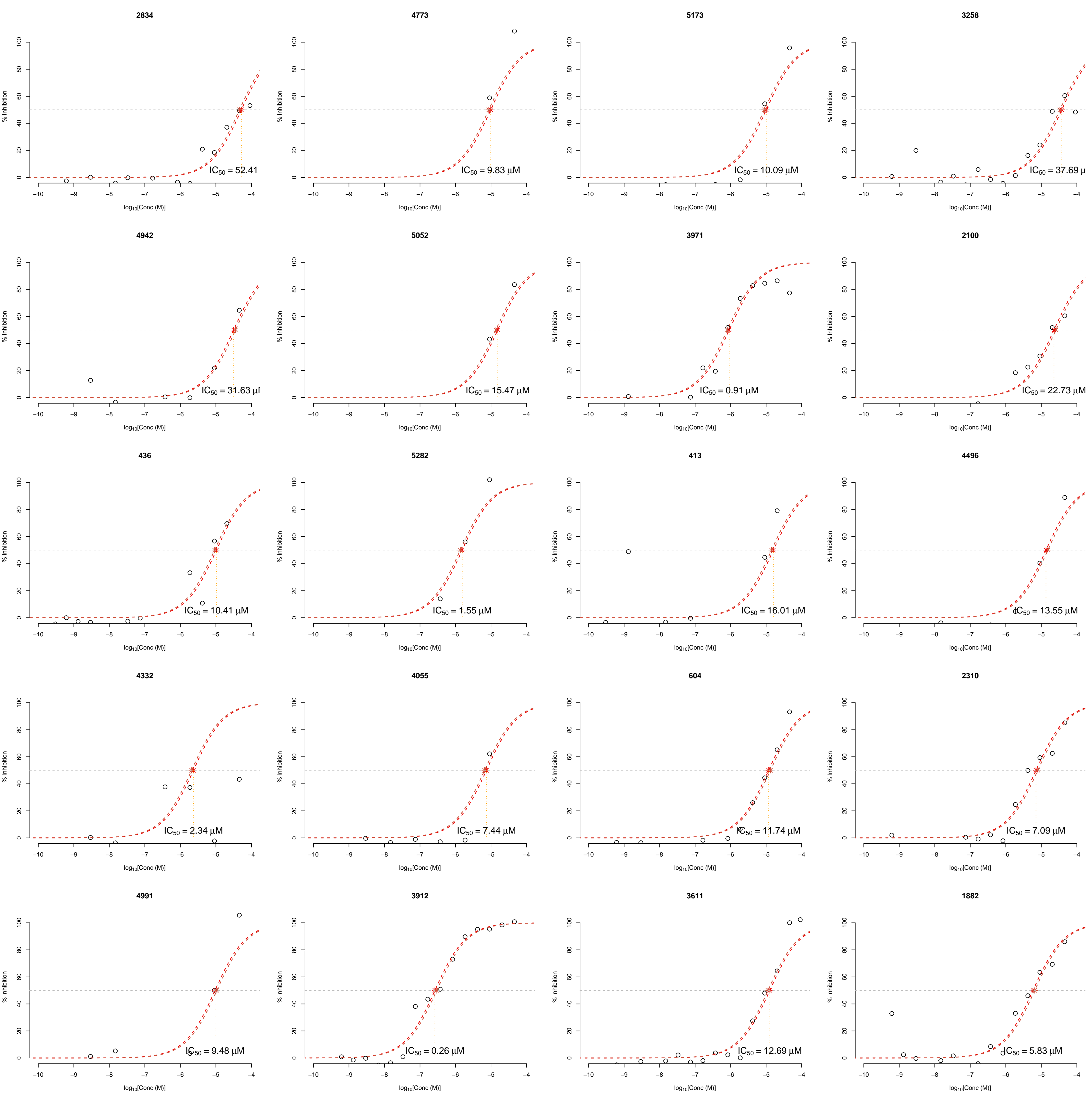

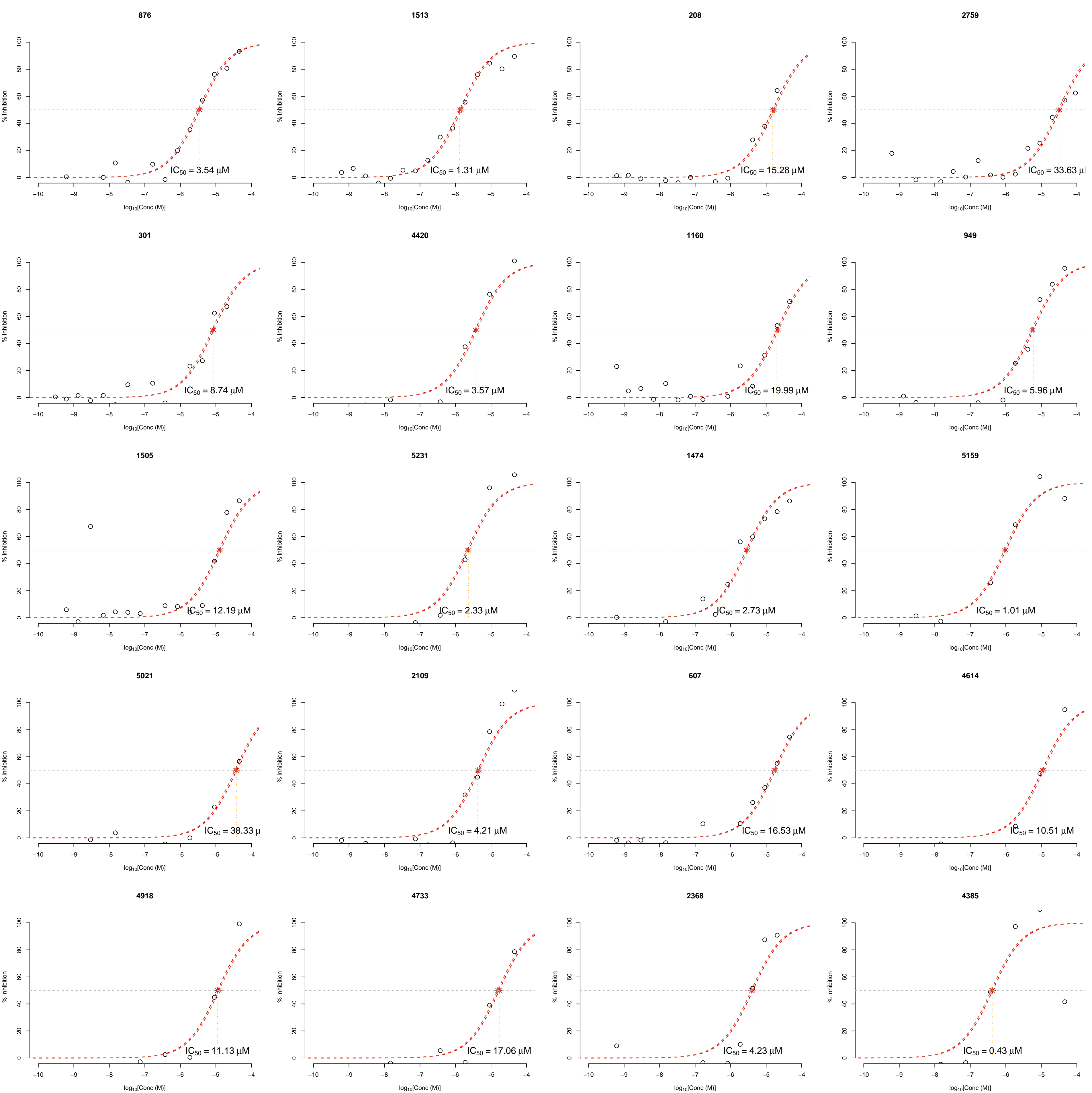

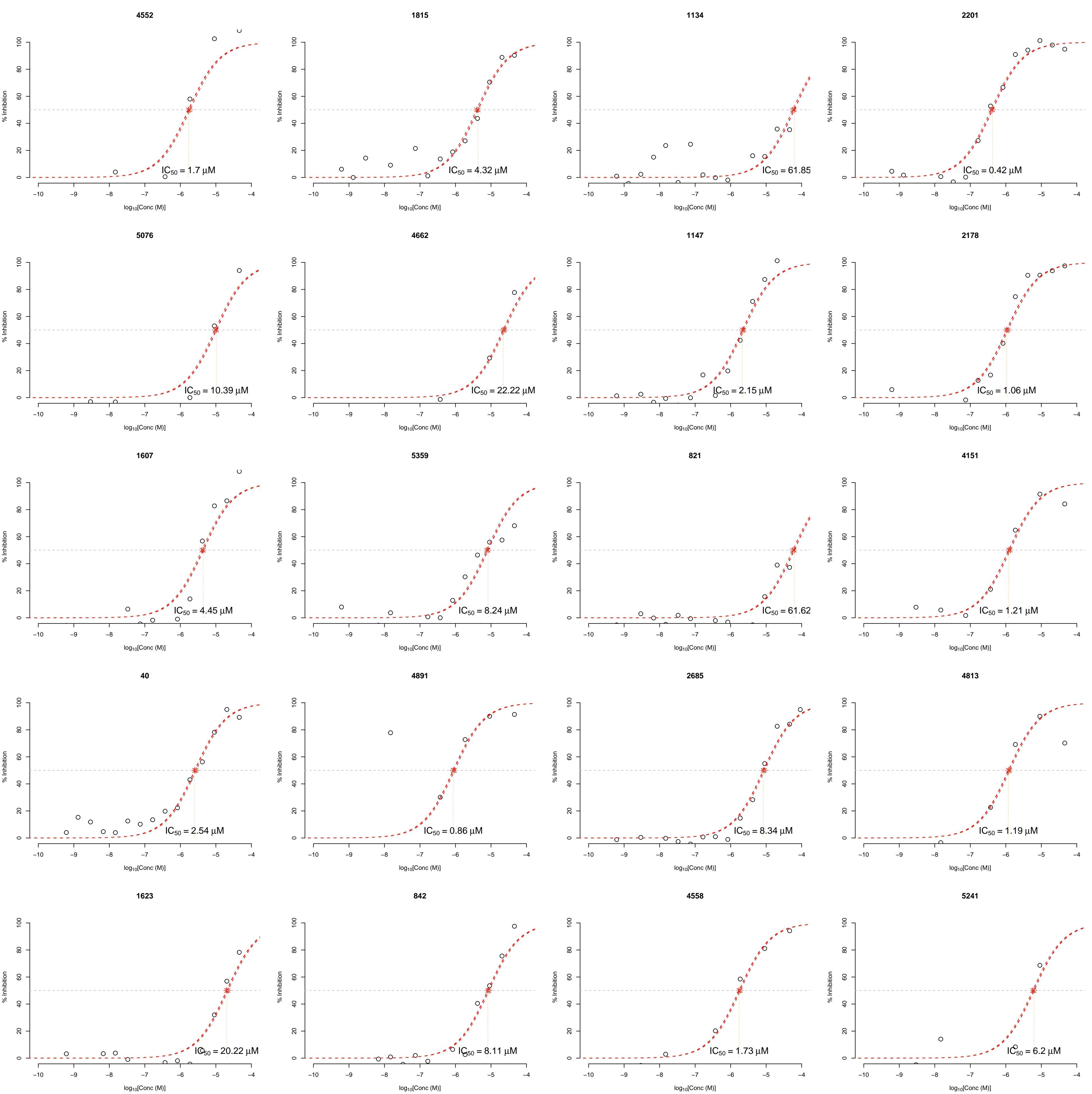

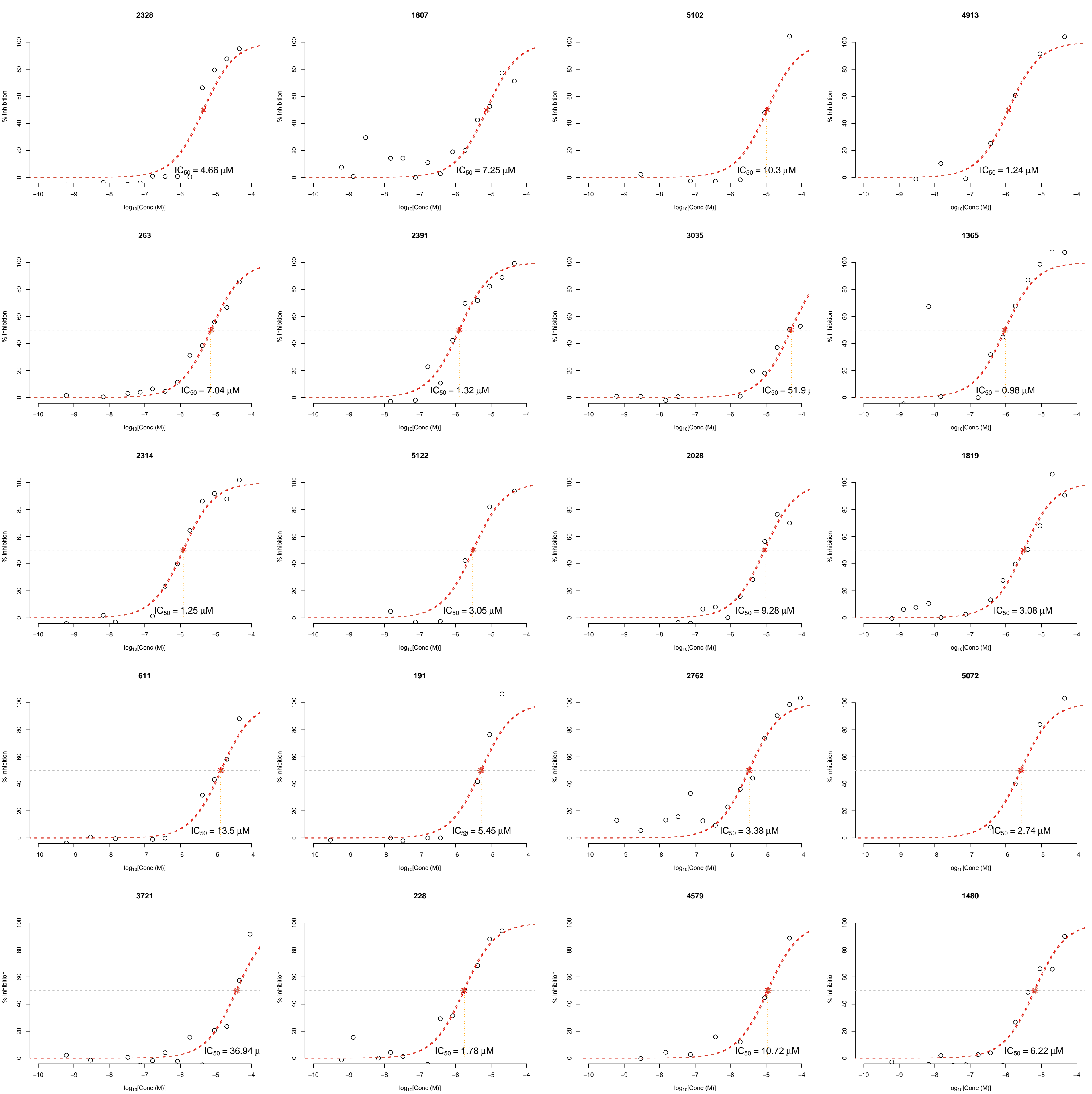

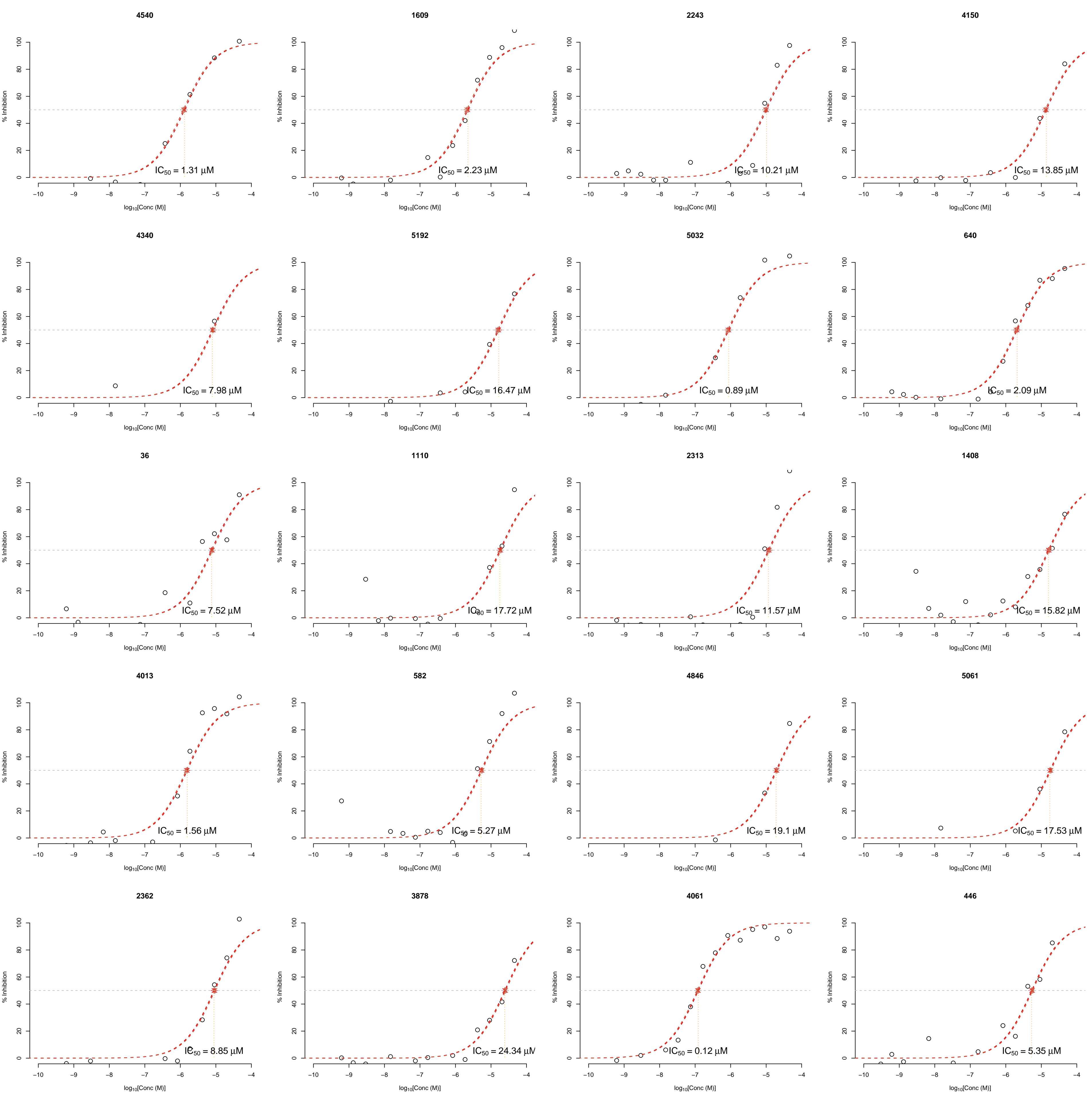

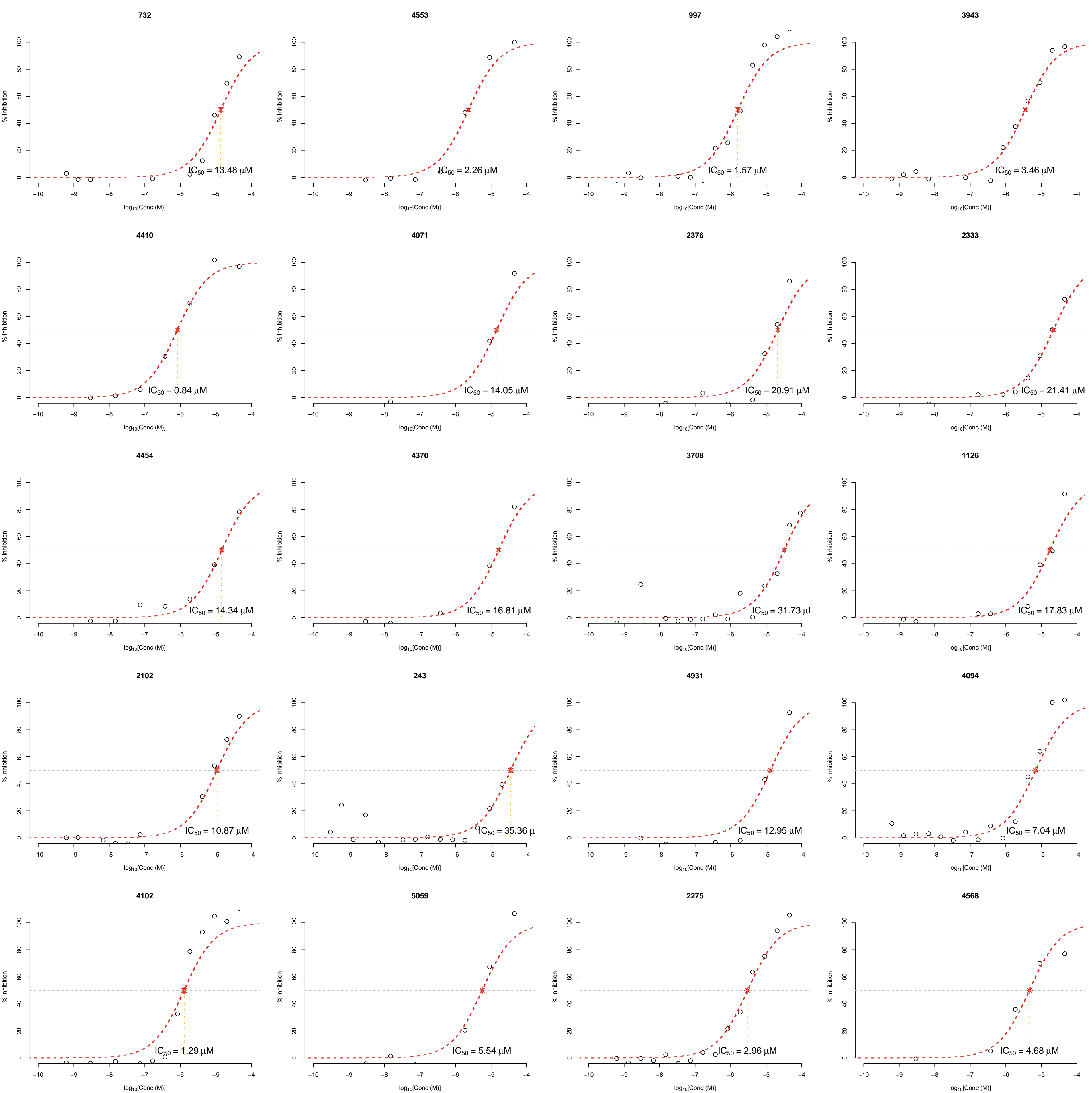

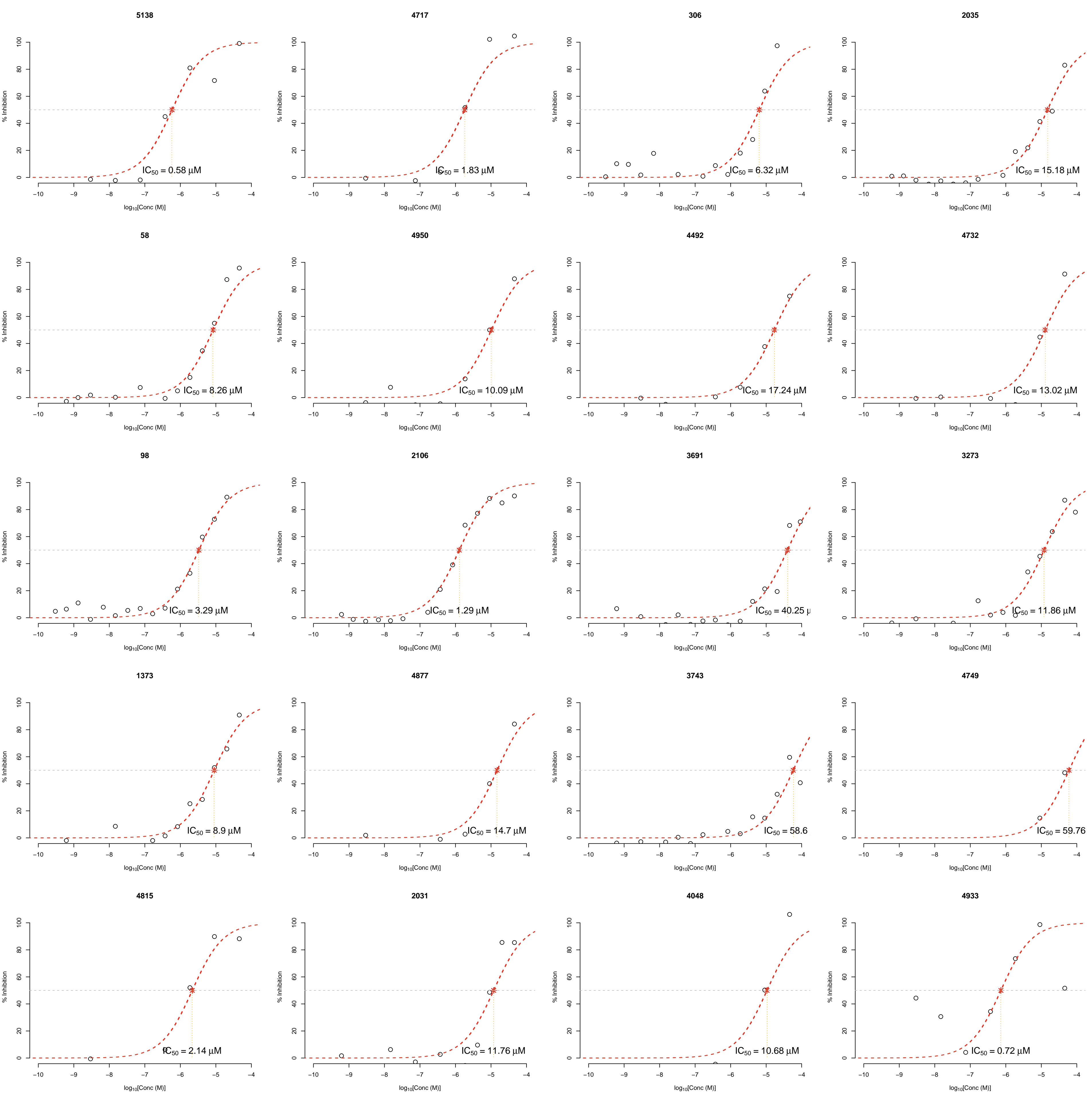

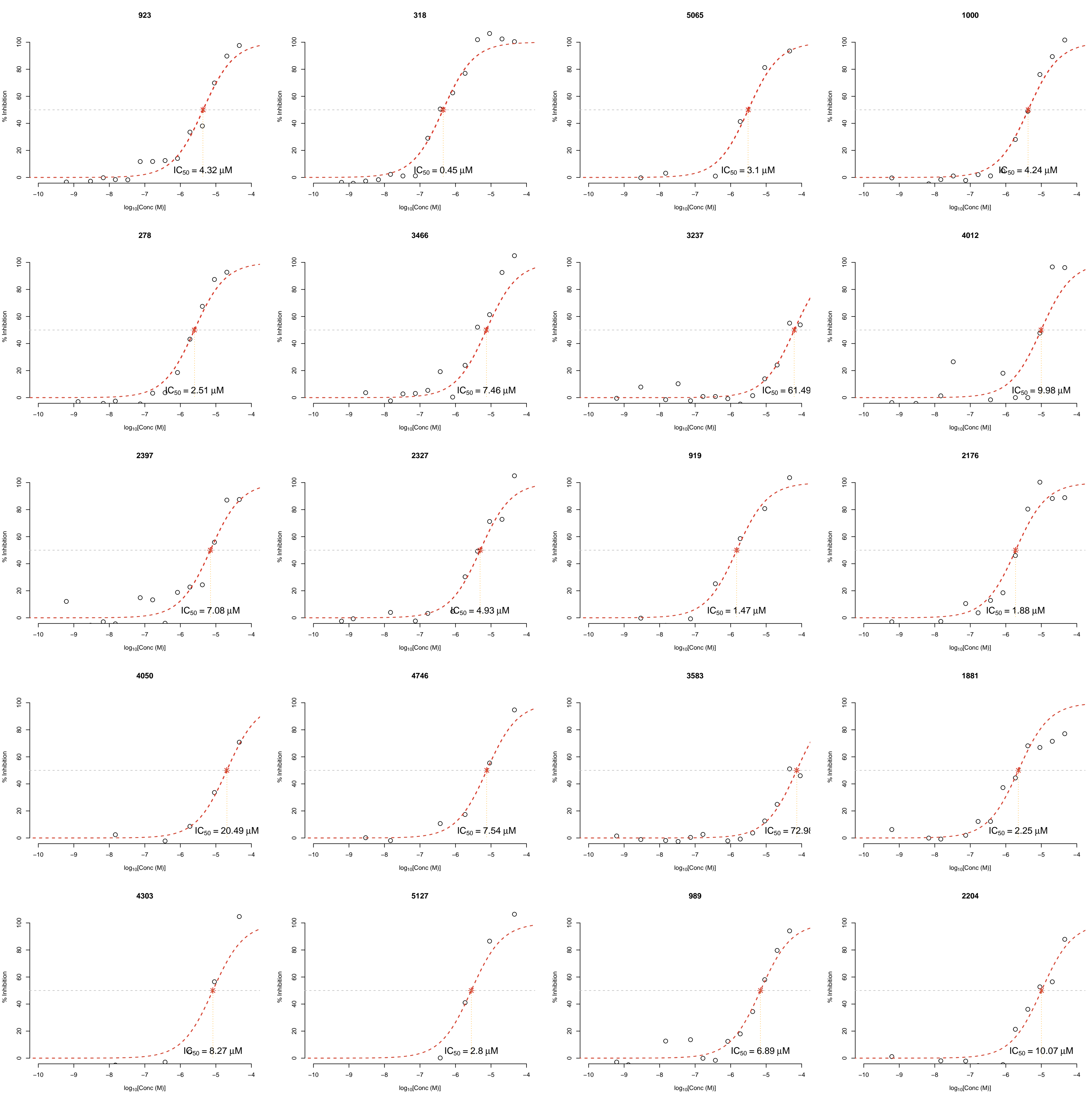

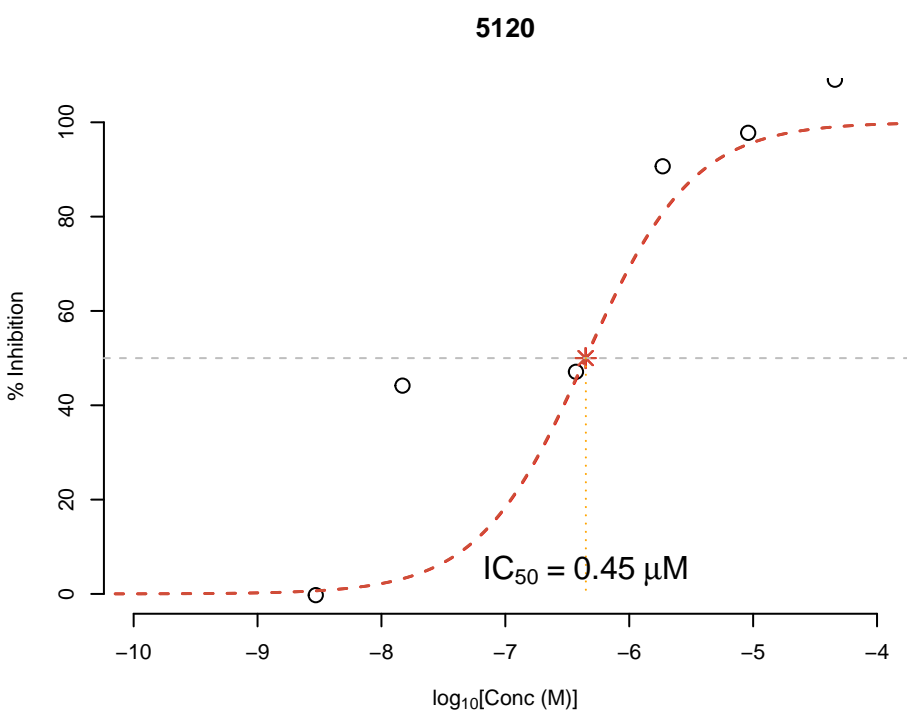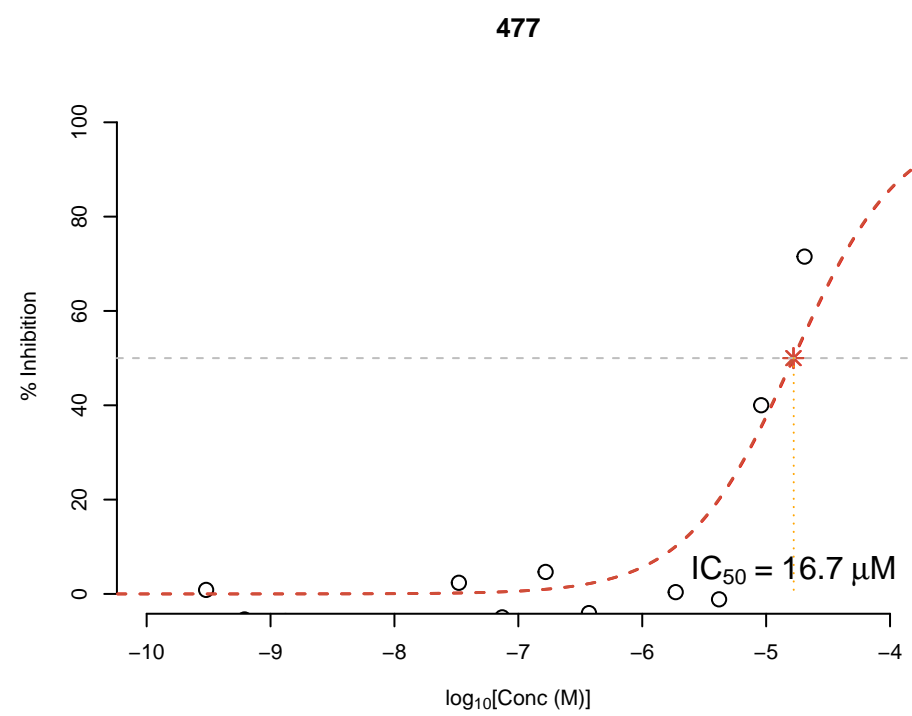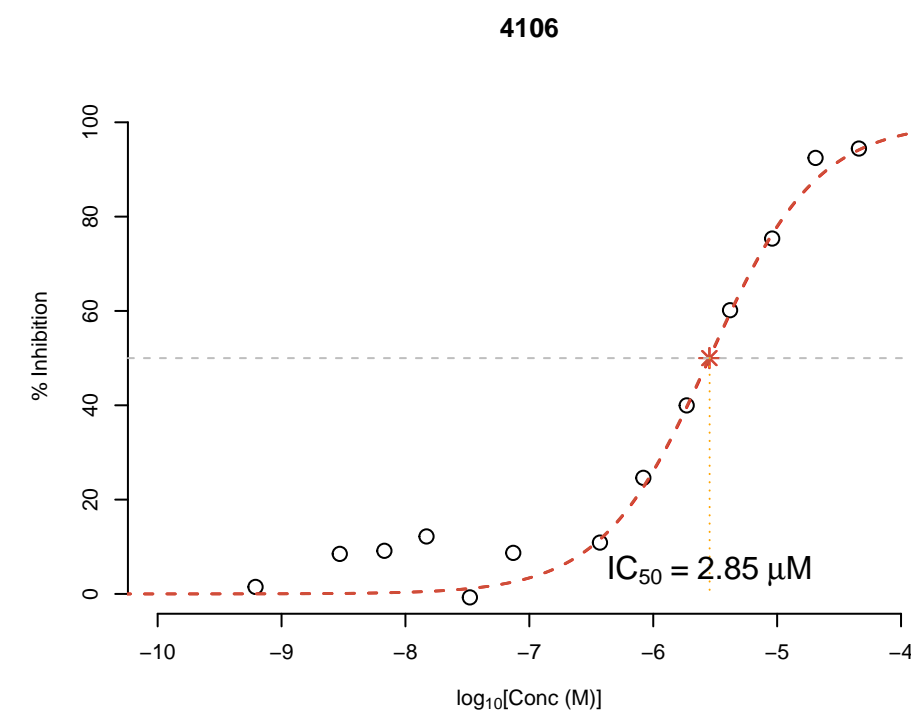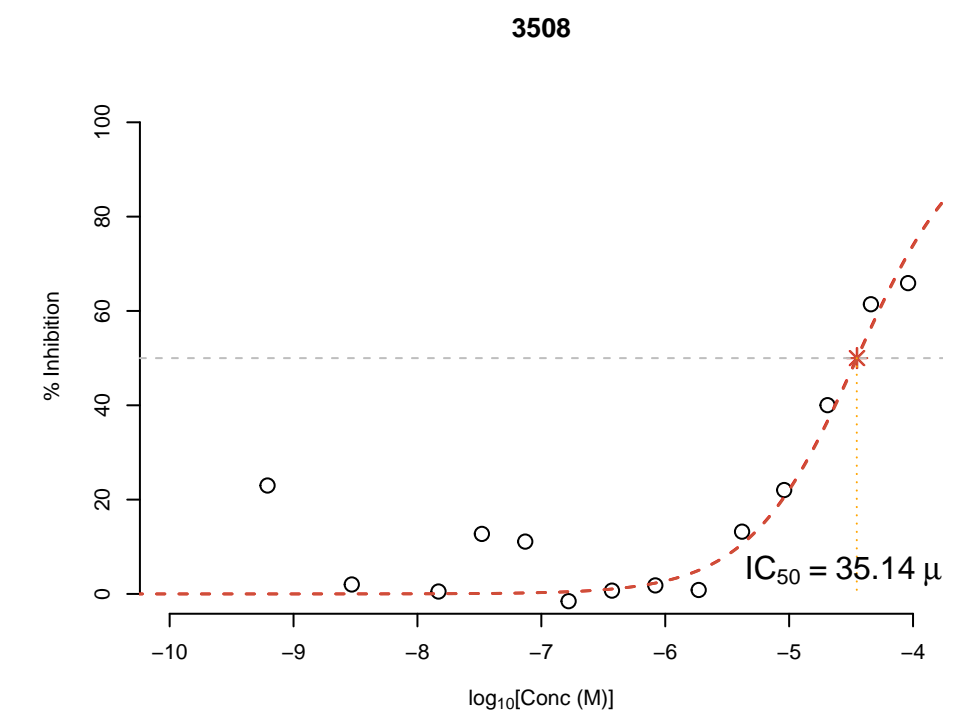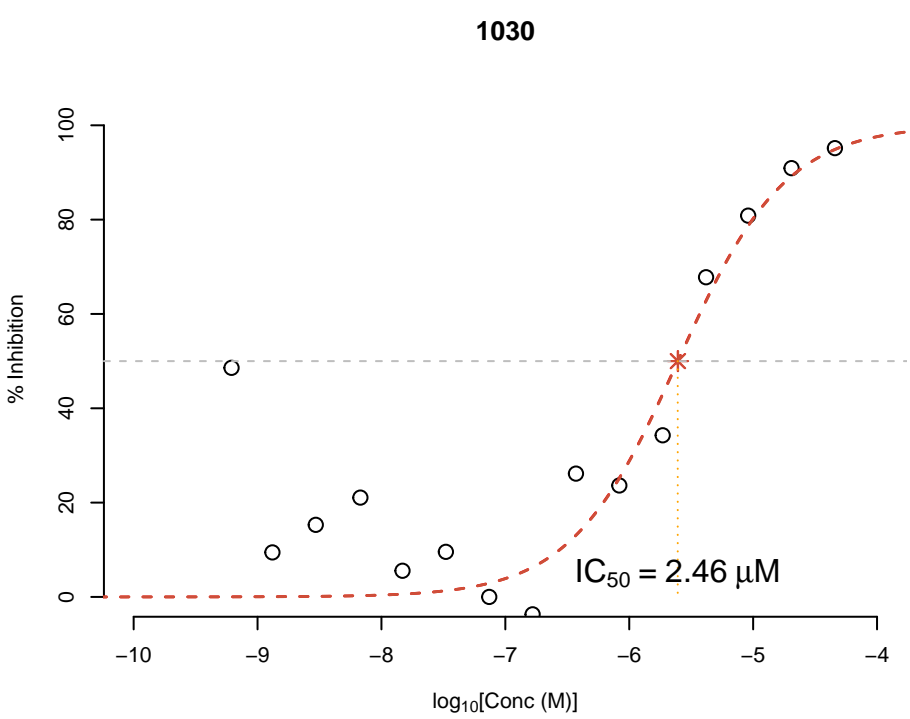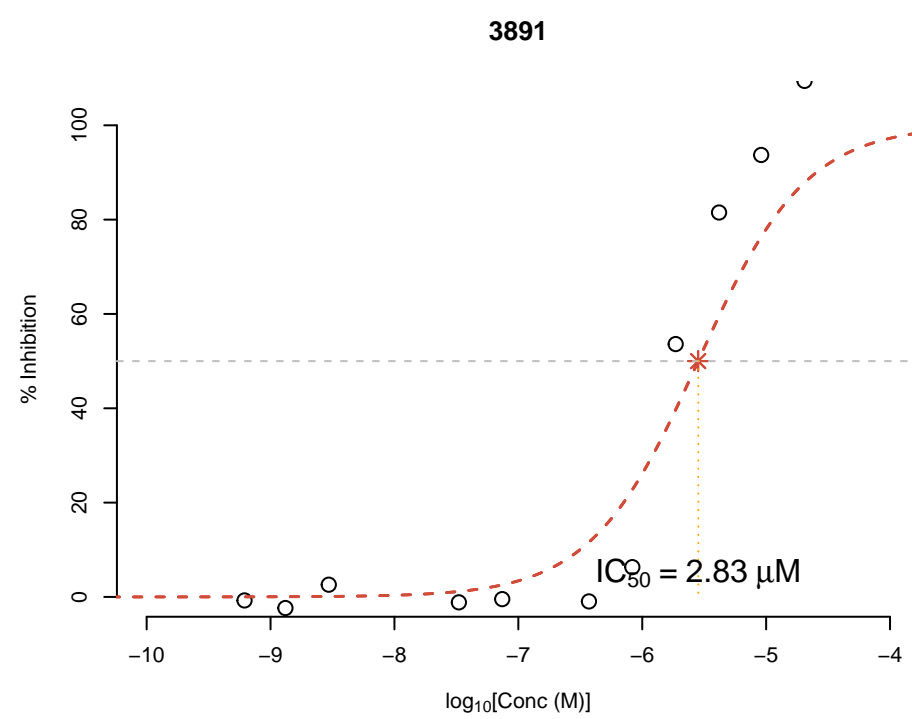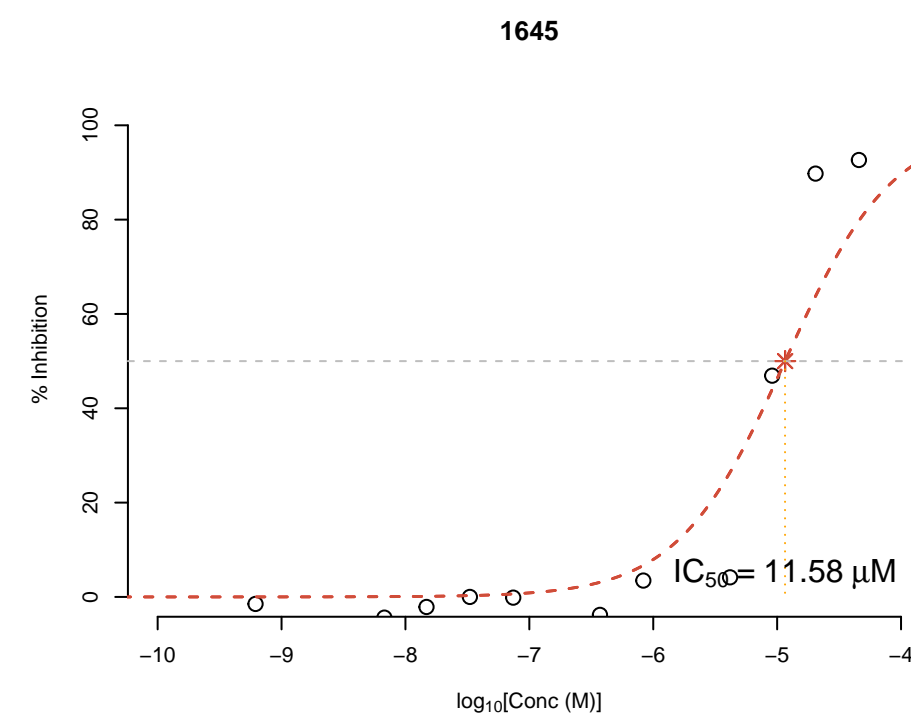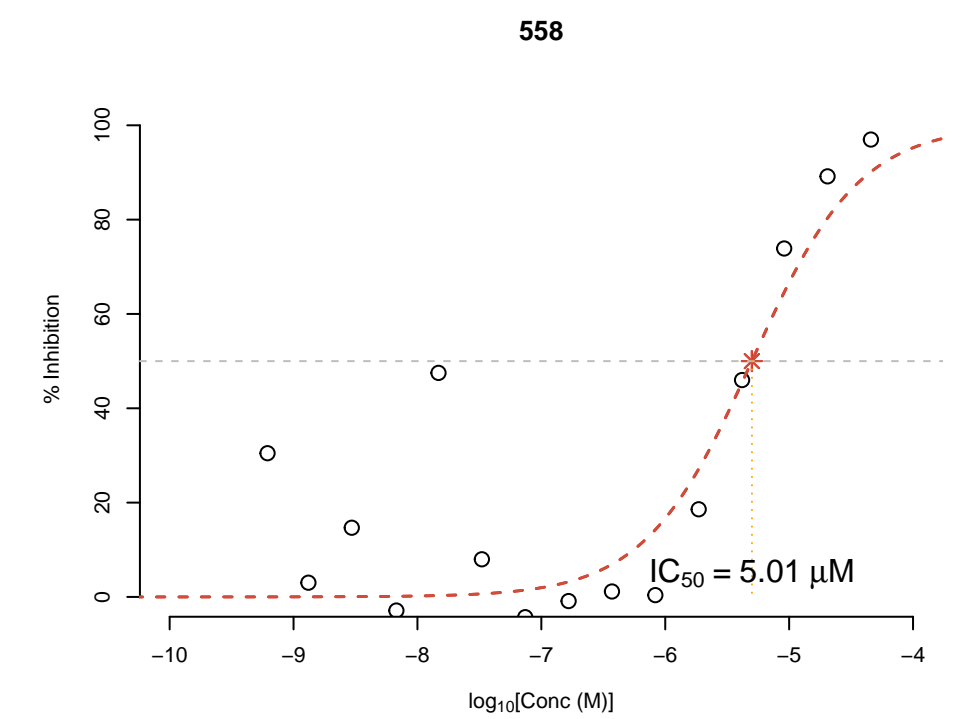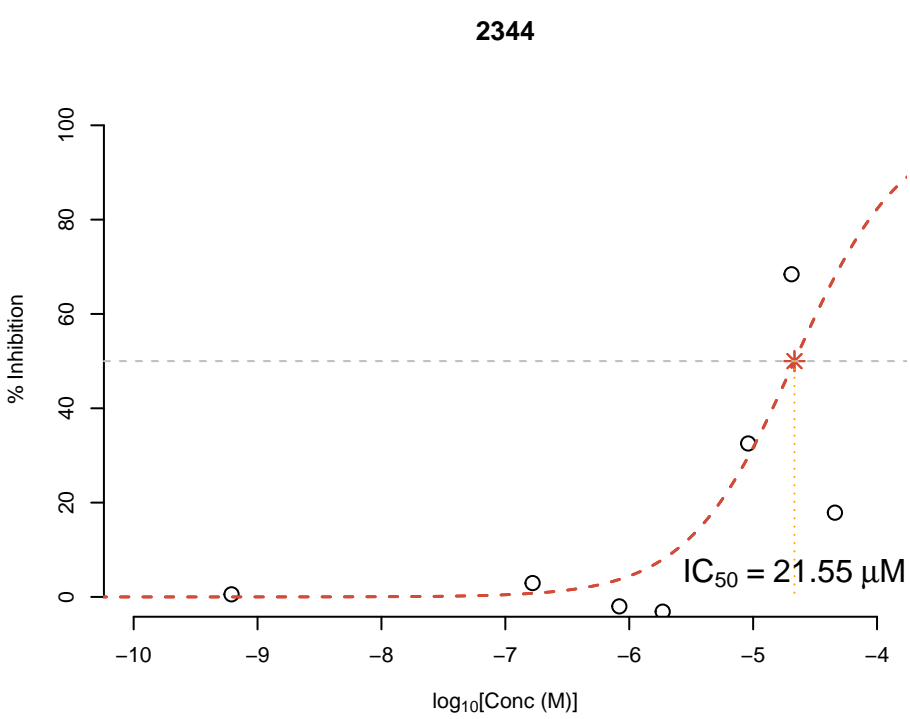

Supplement: Supplementary file 1 [file ijms-24-00635-s001.zip › Figure S1.pdf]
